# Proceedings of the International Cancer Imaging Society Meeting and 20th Annual Teaching Course: ONLINE, 19 - 22 September 2021

**DOI:** 10.1186/s40644-021-00422-6

**Published:** 2021-09-17

## A1 Tumour genomics: primer for radiologists – 13:10-13:30

### Helen Hanson^1,2^ (helen.hanson@stgeorges.nhs.uk)

#### ^1^South West Thames Regional Genetics Service, St Georges Hospital’s NHS Foundation Trust; ^2^Institute of Cancer Research, London

Integration of genomic data into care of cancer patients is becoming routine practice and knowledge of cancer genomics is important for all radiologists. This talk will aim to orientate attendees to the clinically relevant information that can be obtained from the two genomes of cancer patients: the somatic (tumour) genome and the germline (constitutional) genome with specific focus on the relevance for radiologists.

## A2 Genetic insights into screening for prostate cancer - 13:50-14:10

### Elizabeth K Bancroft^1,2^, Jana McHugh^1,2^, Ros A Eeles^1,2^.

#### ^1^Oncogenetics Team, Institute of Cancer Research, London, UK; ^2^Cancer Genetics Unit, Royal Marsden NHS Foundation Trust, London, UK

##### Correspondence: Elizabeth K Bancroft (elizabeth.bancroft@rmh.nhs.uk)

**Background**

The Oncogenetics Team at the Royal Marsden NHS Foundation Trust / Institute of Cancer Research have a programme of research evaluating the integration of germline genomics into the prostate cancer screening and diagnostic pathway. Genetic factors have been identified that contribute to the development of prostate cancer, disease aggressiveness and effectiveness of different treatments.

There is great potential for using genetics to stratify people into different risk categories to enable screening and prevention measures to be targeted at those most at risk. In prostate cancer, this approach has potential to detect clinically significant cancers at an early stage, minimising the social and economic impact for both the patient (in terms of time off work and quality of life) and for the health service (targeting resources at those needing them most).

We are running three prostate cancer screening studies for men in different high-risk groups; 1. The IMPACT study (https://clinicaltrials.gov/ct2/show/NCT00261456) for men with pathogenic variants in BRCA1/2 or the mis-match repair genes, (2) the PROFILE study (https://clinicaltrials.gov/ct2/show/NCT02543905) for men with either (i) a family history of prostate cancer or (ii) men of Black African ancestry, (3) The BARCODE1 study (https://www.clinicaltrials.gov/ct2/show/NCT03857477) for men from the general population who have been identified to be in the highest risk group based on a polygenic risk score.

**Materials and methods**

An overview of each of the three screening studies will be provided to describe the participants, screening algorithms and genetic testing processes.

**Results**

The interim results of the IMPACT study will be shown that demonstrate annual prostate screening using a Prostate Specific Antigen (PSA) threshold of 3.0ng/ml detects clinically significant cancers in men with pathogenic variants in *BRCA2* [1], *MSH2* and *MSH6*. The results of the pilot BARCODE1 study (300 men) will be presented to demonstrate the feasibility of this population-based study, summarising uptake rates and cancer incidence [2]. An update on the PROFILE study progress will also be presented.

**Conclusions**

Targeting prostate cancer screening at men at highest genetic risk is a feasible and acceptable approach and has potential to detect clinically important disease in specific high-risk groups.

**Acknowledgements**

The IMPACT study Steering Committee and Collaborators, the PROFILE study Steering Committee and Collaborators and BARCODE1 Steering Committee and Collaborators.

**References**

[1] Page EC, Bancroft EK, Brook MN et al. Interim Results from the IMPACT Study: Evidence for Prostate-specific Antigen Screening in BRCA2 Mutation Carriers. Eur Urol 2019 Dec;76(6):831-842. doi: 10.1016/j.eururo.2019.08.019.

[2] Benafif S, Ni Raghallaigh H, McGrowder E et al The BARCODE1 Pilot: a feasibility study of using germline SNPs to target prostate cancer screening. British Journal of Urology International (in Press) BJU-2020-2170.R2

## A3 Extra-visceral soft tissue masses in the retroperitoneum and abdomen – 15:50-16:20

### Christina Messiou (Christina.Messiou@rmh.nhs.uk)

#### Department of Radiology, The Royal Marsden Hospital, London, UK

Case-based teaching will be used to exemplify a method for systematic interrogation of indeterminate masses based on an algorithm developed for the retroperitoneum but which can also be applied to the remainder of the abdomen and abdominal wall [1,2]. Features of common masses and characteristic features of less common aetiologies will be presented through interactive cases.

Extra-visceral soft tissue masses in the retroperitoneum and abdomen can be incidental findings and have a broad differential diagnosis. Although rare, the potential diagnosis of soft tissue sarcoma should be considered early to ensure referral to a soft tissue sarcoma centre and to avoid inappropriate excision biopsy or incomplete resection. Completeness of surgical resection and centralized multidisciplinary management in a specialist sarcoma centre are both key factors influencing long term survival [3,4]. Twenty percent of indeterminate masses referred to sarcoma centres are not sarcomas and imaging diagnosis can be challenging [5]. A tissue diagnosis using core needle biopsy is crucial for differentiating benign from malignant pathologies, sarcoma from non-sarcomas and for treatment planning which may include neoadjuvant therapy for some tumour types. Core needle biopsy is safe and does not influence local recurrence or overall survival for patients with sarcoma [6,7].

Although sarcomas are rare, the most common subtype in adults is liposarcoma and therefore interrogation of an indeterminate mass should begin with a careful search for the presence of fat. If fat is identified, features suggesting benign lesions such as renal angiomyolipoma or adrenal myelolipoma should be considered. Non-fatty masses can reflect metastatic disease or germ cell tumours and therefore complete staging and tumour markers are advised. The second commonest sarcoma in the retroperitoneum is leiomyosarcoma. These can be easily recognised due to their venous origin, usually the inferior vena cava, although renal and gonadal vein leiomyosarcomas are also seen. Presence of lymph nodes usually suggests a non-sarcomatous cause (i.e. lymphoma or metastatic disease) as with the exception of rare subtypes (rhabdomyosarcoma, clear cell, epitheliod sarcoma) nodal disease is rare in sarcoma. Schwannomas are the most common benign soft tissue tumour in the retroperitoneum and are usually well defined, slow growing and can show cystic degeneration. In particular, for patients with NF1, malignant peripheral nerve sheath tumours should also be considered as they are highly aggressive sarcomas with poor outcome.

Masses in the anterior abdominal wall present a specific challenge and benign aetiologies predominate [2]. In younger females, fibromatosis is the commonest cause, followed by lipomas and inflammation. Malignant masses in the anterior abdominal wall are frequently metastases but sarcomas and lymphoma should also be considered.

**References**

1. Messiou C, Moskovic E, Vanel D et al. Primary retroperitoneal soft tissue sarcoma: Imaging appearances, pitfalls and diagnostic algorithm. Eu J Surg Onc 2017;43:1191-1198.

2. Bashir U, Moskovic E, Strauss D et al. Soft-tissue masses in the abdominal wall. Clin Rad 2014;69:422-31.

3. Messiou C, Morosi C. Imaging in retroperitoneal sarcomas. J Surg Oncon 2018; 117:25-32.

4. van Houdt W, Zaidi S, Messiou C et al. Treatment of retoperitoneal sarcoma: current standards and new developments. Curr Opin Oncol 2017;29: 260-267.

5. Morosi C, Stacchiotti S, Marcchiano A et al. Correlation between radiological assessment and histopathological diagnosis in retroperitoneal tumors: analysis of 291 consecutive patients at a tertiary reference sarcoma center. Eu J Surg Oncol 2014;40:1662-70.

6. Wilkinson M, Martin J, Khan A et al. Percutaneous core needle biopsy in retroperitoneal sarcomas does not influence local recurrence or overall survival. Ann Surg Oncol 2015;22:853-858.

7. Strauss D, Qureshi YA, Hayes AJ et al. The role of core needle biopsy in the diagnosis of suspected soft tissue tumours. J Surg Oncol 2010;102:523-9.

## A4 Pancreatic cystic lesions – 17:00-17:20

### Giovanni Morana, Silvia Venturini

#### Radiological Department, General Hospital Ca’ Foncello, Treviso (Italy)

##### Correspondence: Giovanni Morana (giovanni.morana@aulss2.veneto.it)

Cystic pancreatic lesions (CPLs) are frequently detected in daily practice, with a detection range from 2.4 % to 19.6 %, whose prevalence as well as size and number increase with age (from 7.9 below 70 y to 40.2 over 70 y) [1–5]. A precise characterization is fundamental for the correct management of these lesions, as they have heterogeneous biological behavior and different prognosis (according to histological type and differentiation) thus requiring different therapeutic options [6].

According to data extrapolated from WHO classification, CPLs are classified in epithelial and non-epithelial and each of these categories are further subdivided in neoplastic and non-neoplastic [7]

From the radiological viewpoint, the key factor to characterize a CPL is to establish whether the lesion communicates or not with the main pancreatic duct (MPD) (Tab. 1).

**Table:** 

**Cystic Pancreatic Lesions: radiological classification**		
**Non-communicating with MPD**	**Non-neoplastic**	WON Congenital cyst Retention Cyst
	**Neoplastic**	Mucinous - Mucinous cystic neoplasms (MCN) Non mucinous - Serous cystic neoplasm (SCN) - Solid pseudopapillary neoplasm (SPN) - Cystic pancreatic neuroendocrine neoplasms (CPNETs) - Acinar cell cystic neoplasm (ACCN) Ductal adenocarcinoma with cystic degeneration
**Communicating with MPD**	**Non-neoplastic**	Pseudocyst WON
	**Neoplastic**	Intraductal papillary mucinous neoplasm (IPMN)

Tab. 1 Radiological classification of CPLs.

The role of imaging is to differentiate benign from malignant or potentially malignant CPLs avoiding unnecessary surgery and to early detect morphological changes in potentially malignant CPLs in order to offer more chance of survival to these patients.

Both MRI with MRCP and MDCT have high diagnostic performance in differentiating benign from malignant CPLs, with an accuracy ranging from 73% to 81% for MRI and 75% to 78% for MDCT, respectively [8–11].

With Imaging, best with MRI, it is possible to classify the different lesions, to highlight the imaging features suggestive for the pathological diagnosis, to identify the imaging criteria suggesting the malignant evolutions of CPLs, thus addressing the patient to the best therapeutic option.

As a rule of thumb, after excluding pseudocyst or WON (in case of lack of history of severe acute pancreatitis) [12], most frequent non communicating CPLs can be mucinous or serous cystadenomas, whose differential diagnosis is important due to the different management of these lesions, surgical for the first, while most frequent communicating CPLs are given by IPMNs, where it is important to detect worrisome features or high risk stigmata, which require a strict follow-up or surgical resection if patient is fit [13].

However, in many cases the precise nature of a CPL remains indeterminate. Different guidelines will be offered in order to best manage also indeterminate CPLs [7, 14].

The management of CPLs is deeply supported by Imaging, however also clinical criteria (clinical history, habits, age, sex, comorbidities, etc) and other diagnostic tools (EUS also with biopsy, laboratory findings) are important for a correct management of CPLs, thus their management is best obtained with a multidisciplinary approach.

References

1. Zhang X-M, Mitchell DG, Dohke M, et al (2002) Pancreatic cysts: depiction on single-shot fast spin-echo MR images. Radiology 223:547–553. https://doi.org/10.1148/radiol.2232010815

2. Ks S, Te F, Ra D, et al (2004) Cystic pancreatic neoplasms: observe or operate. In: Ann. Surg. https://pubmed.ncbi.nlm.nih.gov/15082969/. Accessed 10 Feb 2021

3. Laffan TA, Horton KM, Klein AP, et al (2008) Prevalence of unsuspected pancreatic cysts on MDCT. AJR Am J Roentgenol 191:802–807. https://doi.org/10.2214/AJR.07.3340

4. de Jong K, Nio CY, Hermans JJ, et al (2010) High prevalence of pancreatic cysts detected by screening magnetic resonance imaging examinations. Clin Gastroenterol Hepatol Off Clin Pract J Am Gastroenterol Assoc 8:806–811. https://doi.org/10.1016/j.cgh.2010.05.017

5. Ks L, A S, Nm R, I P (2010) Prevalence of incidental pancreatic cysts in the adult population on MR imaging. In: Am. J. Gastroenterol. https://pubmed.ncbi.nlm.nih.gov/20354507/. Accessed 8 Feb 2021

6. Brugge WR (2015) Diagnosis and management of cystic lesions of the pancreas. J Gastrointest Oncol 6:375–388. https://doi.org/10.3978/j.issn.2078-6891.2015.057

7. European Study Group on Cystic Tumours of the Pancreas (2018) European evidence-based guidelines on pancreatic cystic neoplasms. Gut 67:789–804. https://doi.org/10.1136/gutjnl-2018-316027

8. Ni S, A S, V D, et al (2009) Comparative performance of MDCT and MRI with MR cholangiopancreatography in characterizing small pancreatic cysts. In: AJR Am. J. Roentgenol. https://pubmed.ncbi.nlm.nih.gov/19696285/. Accessed 8 Feb 2021

9. Hj L, Mj K, Jy C, et al (2011) Relative accuracy of CT and MRI in the differentiation of benign from malignant pancreatic cystic lesions. In: Clin. Radiol. https://pubmed.ncbi.nlm.nih.gov/21356393/. Accessed 8 Feb 2021

10. Jh K, Hw E, Hj P, et al (2012) Diagnostic performance of MRI and EUS in the differentiation of benign from malignant pancreatic cyst and cyst communication with the main duct. In: Eur. J. Radiol. https://pubmed.ncbi.nlm.nih.gov/22227264/. Accessed 8 Feb 2021

11. Sahani DV, Sainani NI, Blake MA, et al (2011) Prospective evaluation of reader performance on MDCT in characterization of cystic pancreatic lesions and prediction of cyst biologic aggressiveness. AJR Am J Roentgenol 197:W53-61. https://doi.org/10.2214/AJR.10.5866

12. Banks PA, Bollen TL, Dervenis C, et al (2013) Classification of acute pancreatitis--2012: revision of the Atlanta classification and definitions by international consensus. Gut 62:102–111. https://doi.org/10.1136/gutjnl-2012-302779

13. Tanaka M (2019) Clinical Management and Surgical Decision-Making of IPMN of the Pancreas. In: Methods Mol. Biol. Clifton NJ. https://pubmed.ncbi.nlm.nih.gov/30378040/. Accessed 23 Feb 2021

14. Megibow AJ, Baker ME, Morgan DE, et al (2017) Management of Incidental Pancreatic Cysts: A White Paper of the ACR Incidental Findings Committee. J Am Coll Radiol JACR 14:911–923. https://doi.org/10.1016/j.jacr.2017.03.010

## A5 Cystic Renal Masses: An update on Bosniak classification – 17:20-17:40

### Hersh Chandarana (hersh.chandarana@nyumc.org)

#### Department of Radiology, New York University Grossman School of Medicine, New York, NY, USA

A classification and management system for cystic renal lesions (initially on CT imaging) was proposed by Dr. Bosniak nearly 35 years ago [1]. This imaging and management classification system is commonly used to evaluate cystic renal lesions. This has had a major impact in decreasing over-treatment of cystic renal lesions [2].

The classification and management system can be briefly summarized as follows:

Category 1: Simple cyst (Management: No follow-up or intervention)

Category 2: Benign cyst with thin septation or calcification (Management: No follow-up or intervention).

Category 2F: Mildly complicated cysts (Management: Need follow-up to exclude malignancy)

Category 3: Indeterminate cystic lesions (Management includes surgical resection)

Category 4: Cystic RCC (Management is usually surgical)

However, there are number of challenges of the original imaging classification such as (1) system is subjective and lacks definition for various terms such as thick or thin wall and septa, resulting in inter-reader variability; (2) does not fully account for our current understanding of the natural history of cystic renal cell cancer (RCC) which are indolent and less aggressive compared to the solid renal lesions. Furthermore, considerable proportion of Category 3 lesions tend to be benign at pathology. These patients if managed conservatively would have benefited from not undergoing surgical resection [3-5].

Revised Bosniak Classification (v2019) was proposed in 2019 [6]. The goal of this revised system was to improve specificity and decrease false positive in the patients that undergo surgery for suspected cystic renal cancer, reduce inter-reader variability by defining and clarifying various terms, provide quantitative definition of certain terms (such as thin and thick septa and wall), and thus help further decrease over diagnosis and over treatment of cystic renal lesions. Number of recent papers have elaborated on these revisions [7], but some of the substantive changes include:

- Defining cystic lesions as having less than 25% solid enhancing component
- Defining enhancement
- Defining wall and septal thickness
- Defining few (1-3) and many (>3) septa
- Expanding the definition of Bosniak Type 2 cysts and benign lesions

Recent studies suggest that the revised system has potential to decrease over diagnosis and over-treatment of cystic renal lesions [8-10].

References:

1. Bosniak MA. The current radiological approach to renal cysts. Radiology. 1986 Jan;158(1):1-10

2. Bosniak MA. The Bosniak renal cyst classification: 25 years later. Radiology. 2012 Mar;262(3):781-5.

3. Schoots IG, Zaccai K, Hunink MG, et al. Bosniak Classification for Complex Renal Cysts Reevaluated: A Systematic Review. J Urol. 2017 Jul;198(1):12-21.

4. Siegel CL, McFarland EG, Brink JA, et al. CT of cystic renal masses: analysis of diagnostic performance and interobserver variation. AJR Am J Roentgenol 1997;169(3):813–818.

5. Smith AD, Carson JD, Sirous R, et al. Active surveillance versus nephronsparing surgery for a Bosniak IIF or III renal cyst: a cost-effectiveness analysis. AJR Am J Roentgenol 2019;212(4):830–838.

6. Silverman SG, Pedrosa I, Ellis JH, et al. Bosniak Classification of Cystic Renal Masses, Version 2019: An Update Proposal and Needs Assessment. Radiology. 2019 Aug;292(2):475-488.

7. Schieda N, Davenport MS, Krishna S, et al. Bosniak Classification of Cystic Renal Masses, Version 2019: A Pictorial Guide to Clinical Use. Radiographics. May-Jun 2021;41(3):814-828.

8. Yan JH, Chan J, Osman H, et al. Bosniak Classification version 2019: validation and comparison to original classification in pathologically confirmed cystic masses. Eur Radiol. 2021 May 21.

9. Bai X, Sun S, Xu W, et al. MRI-based Bosniak Classification of Cystic Renal Masses, Version 2019: Interobserver Agreement, Impact of Readers' Experience, and Diagnostic Performance. Radiology. 2020 Dec;297(3):597-605.

10. Tse JR, Shen L, Shen J, et al. Prevalence of Malignancy and Histopathological Association of Bosniak Classification, Version 2019 Class III and IV Cystic Renal Masses. J Urol. 2021Apr;205(4):1031-1038.

## A6 Clinical applications of new MRI technologies in oncology – 13:40-14:00

### Erica D Scurr (Erica.Scurr@rmh.nhs.uk)

#### The Royal Marsden NHS Foundation Trust, London and Surrey, UK

Due to its superior soft tissue contrast MRI is the primary imaging modality for the characterisation and staging of disease. However, to achieve the desired image contrast and coverage, MRI acquisitions are relatively slow, and body imaging can be corrupted with artifacts due to patient movement, as well as involuntary cardiac, respiratory, and peristaltic motion. MR acquisitions can be synchronised to regular cardiac and/or respiratory motion but at the expense of further increases in acquisition time. Therefore, both academic researchers and the MRI system vendors are constantly striving to develop new MR acquisition methods that can reduce overall acquisition times without significantly affecting image quality, as well as developing methods that are intrinsically robust to motion.

Many of these methods have been driven by improvements in the MRI system hardware performance. Improvements in gradient technology allow for improved imaging efficiency, whilst the development of high channel count coil arrays enables faster imaging through the mathematical combination of signals from individual coil elements.

The initial breakthrough in imaging speed was the commercialisation of the RARE technique, developed by Jurgen Hennig in the late 1980s, as the fast or turbo spin echo (FSE or TSE) method [1]. This allowed multiple “lines” of raw data to be acquired in a single repetition time (TR) substantially reducing the acquisition time for T2-weighted imaging.

The new millennium saw the introduction of partially parallel imaging methods, such as SENSE and GRAPPA, where the spatially varying signal sensitivities of the multiple elements making up each receive coil array could be used to reconstruct images where the raw data was sub-sampled resulting in further reductions in acquisition times across most imaging sequences. These hardware and software developments bought the acquisition times of 3D gradient-echo imaging methods, with whole organ coverage, down to the duration of a single breath-hold.

Non-Cartesian MRI acquisitions, such as projection reconstruction imaging have been resurrected and introduced as 3D acquisitions known as ‘stack-of-stars’[2]. They have the advantage that the centre of the raw image space is repeatedly sampled, this provides an intrinsic motion averaging allowing high quality images to be obtained during free breathing.

Further reductions in acquisition time were first demonstrated in 2007 by Miki Lustig who applied the concept of compressed sensing (CS) to MR image acquisition and reconstruction. CS is based upon pseudo-random sub-sampling of the MR raw data, followed by an iterative reconstruction algorithm [3,4]. CS provides the greatest acceleration when applied to 3D acquisitions where data in both phase encoding directions can be subsampled, or in dynamic 2D/3D imaging where the subsampling can also be applied with time.

Reductions in multi-slice 2D image acquisition time have also been achieved using simultaneous multi-slice (SMS) imaging [5], in which specially crafted RF excitation pulses are used to excite several slices simultaneously and the spatial sensitivities of the receive coil array is used to mathematically separate the slices.

This presentation will explain the physical principles of these imaging methods and demonstrate the advantages and challenges of applying them in the clinic.

**References**

1. Hennig J, Nauerth A, Friedburg H. RARE imaging - a fast imaging method for clinical MR. Magnetic Resonance in Medicine 1986; 3: 823-833.
2. Chandarana H, Block TK, Rosenkrantz AB, et al. Free-breathing radial 3D fat-suppressed T1-weighted gradient echo sequence: a viable alternative for contrast-enhanced liver imaging in patients unable to suspend respiration. Investigative Radiology;46:648–653 (2011)
3. Lustig M, Donoho D, Pauly J. Sparse MRI: The Application of Compressed Sensing for Rapid MR Imaging Magnetic Resonance in Medicine 58:1182–1195 (2007)
4. Feng L, Grimm R, Block KT, et al. StarVIBE and Stack of Stars implementation for liver imaging
  Golden-angle radial sparse parallel MRI: combination of compressed sensing, parallel imaging, and golden-angle radial sampling for fast and flexible dynamic volumetric MRI. (The "GRASP" technique) Magnetic Resonance in Medicine; 72:707-717 (2014)
5. Barth M, Breuer F, Koopmans P, Norris D, Poser B. Simultaneous Multislice (SMS) Imaging Techniques Magnetic Resonance in Medicine 75:63–81 (2016)

## A7 Contrast media for liver MRI - when, what and how? – 14:00-14:20

### Bachir Taouli (bachir.taouli@mountsinai.org)

#### Department of Diagnostic, Molecular and Interventional Radiology, BioMedical Engineering and Imaging Institute, Icahn School of Medicine at Mount Sinai, New York, NY, USA

Gadolinium based contrast agents (GBCAs) are essential for diagnosis of liver disease and for detection/characterization of benign and malignant liver tumours. GBCAs enable increased SNR and lesion to liver contrast ratios, enabling improved lesion detection. In addition, enhancement patterns enable precise lesion characterization. For liver MRI, it is possible to use extracellular (EC-GBCAs) GBCAs or liver specific GBCAs (such as gadoxetate or gadobenate dimeglumine).

Liver specific GBCAs enable the acquisition of a hepatocyte phase, whereby the liver parenchyma becomes hyperintense on T1-weighted imaging, making hypointense lesions more conspicuous. The advantages and limitations of EC-GBCAs vs liver specific GBCAs will be discussed in specific scenarios such as HCC/primary liver cancer, metastases, and benign lesions. We will also discuss safety of these agents, and their usage in patients with diminished renal function. Some specific up-to-date protocols will also be presented.

## A8 Commonly asked questions about RECIST 1.1: “How to” guide with FAQs and top tips – 15:20-15:40

### A Rockall, A Sohaib

#### Imperial College, London UK

##### Correspondence: A Rockall (a.rockall@imperial.ac.uk)

In 2000, Response Evaluation Criteria for Solid Tumours (RECIST) was introduced to give standardised method for assessing response to treatments in clinical trials. This was updated RECIST to version 1.1 by the RECIST Working Group. The major changes incorporated were fewer number of lesions assessed, measurement criteria for lymph nodes and requirements for response and progression further clarified.

RECIST is now widely employed in clinical as well clinical trial setting. However, there are pitfalls to be aware of is RECIST assessment and its practical application often leads to commonly asked questions. This presentation gives an overview of questions posed and the solution as per the RECIST guidelines.

**Key References**

1. Eisenhauer EA, Therasse P, Bogaerts J, Schwartz LH, Sargent D, Ford R, Dancey J, Arbuck S, Gwyther S, Mooney M, Rubinstein L, Shankar L, Dodd L, Kaplan R, Lacombe D, Verweij J. New response evaluation criteria in solid tumours: revised RECIST guideline (version 1.1). Eur J Cancer. 2009 Jan;45(2):228-47.
2. Schwartz LH, Litière S, de Vries E, Ford R, Gwyther S, Mandrekar S, Shankar L, Bogaerts J, Chen A, Dancey J, Hayes W, Hodi FS, Hoekstra OS, Huang EP, Lin N, Liu Y, Therasse P, Wolchok JD, Seymour L. RECIST 1.1-Update and clarification: From the RECIST committee. Eur J Cancer. 2016 Jul;62:132-7.

## A9 Rectal Cancer: Assessing Neo-Adjuvant Treatment Response – 16:00-16:20

### Kartik S. Jhaveri (kartik.jhaveri@uhn.ca)

#### Mt. Sinai and Womens’ College Hospital, Toronto, Canada

Magnetic resonance imaging (MRI) is the preferred imaging technique for pre-operative staging and management triage in patients with rectal carcinoma. Downstaging via preoperative neoadjuvant chemoradiotherapy (nCRT) with the intent of subsequent curative resection is a commonly utilized treatment pathway care for patients with locally advanced rectal cancer (LARC). Treatment response after CRT can be variable with absolute pathologic complete response (pCR) being observed in only approximately 15–25 %. Digital rectal examination, endoscopy and MRI are typically utilized for the post nCRT assessment. Accurate estimation of treatment response therein is essential towards further management and MRI is being increasingly utilized in this scenario. Post CRT treatment response can be classified with the MRI tumour regression grade (mrTRG) classification systems described by the Magnetic Resonance Imaging and Rectal Cancer European Equivalence Study (MERCURY) study group and European Society of Gastrointestinal and Abdominal Radiology (ESGAR). The goal is to standardise inter-observer treatment response evaluation with MRI via categorical classification into specific tumour response groups. Although these two mrTRG systems do have some commonality, they do have differences in the number of TRG grades as well as what constitutes a good responder. Although direct agreement between MRI tumour regression grade (mrTRG) and pathologic TRG ( pTRG) is low , MRI can be clinically useful in separating good from bad responders .The accuracy of treatment response evaluation by MRI can be improved via several recently discussed approaches. DWI has been reported to increase the diagnostic accuracy of pCR. Although there is also some evidence that measuring change in ADC (ΔADC) and DWI volumetry can be valuable as a biomarker of post CRT response, these do not appear to be utilized commonly in clinical practice. Dynamic contrast enhanced (DCE)-MRI has been investigated with conflicting results and its routine use is not recommended by ESGAR or SAR guidelines. The ideal scheduling of post-nCRT MRI examination depends on the timing of surgery after CRT. Since surgery is typically considered at approximately 6–8 weeks after the completion of nCRT, the MRI examination should be scheduled 6–8 weeks after the completion of CRT, and close to planned surgery. Although widely used MRI has some limitations. Post-CRT inflammation and oedema can result in overestimation of residual cancer. Additionally, underestimation of small volume residual cancer is well known due to extensive post-CRT fibrotic changes. Even though patients with pCR could have excellent outcomes with organ-preserving strategies such as local excision or non-operative management ( watch-and-wait approach), curative resection via total mesolectal excision (TME) or abdominoperineal resection (APR) is the general approach primarily due to the inability to definitively identify pCR after CRT through clinical examination as well as MRI.

## A10 Lung cancer staging –pitfalls and challenges – 16:40-17:00

### Theresa C. McLoud (mcloud.theresa@mgh.harvard.edu)

#### Harvard Medical School, Boston, USA

The TNM staging system approved by the International Association for the Study of Lung Cancer and the American Joint Committee on Cancer was recently revised. The latest edition is the 8th addition published in January 2017. The important changes included a modification of the T classification based on 1 cm increments, downstaging of the T descriptor including endobronchial tumour disregarding its distance from the carina (T2), merging total and partial atelectasis /pneumonia into the same category (T2), upstaging diaphragmatic invasion to T4, introducing the new classification concept of adenocarcinoma in situ and minimally invasive adenocarcinoma into pure and part solid ground glass nodules,and further division of extrathoracic metastases into M1b and M1c based on the number and sites of extrathoracic metastases.

This new TNM staging system provided more precise classification based on prognostic analysis of each of the TNM descriptors. However there are still some potential limitations and clinical situations that have not yet been clarified in the clinical staging determined by imaging. Radiologists need to both understand the changes in staging of the 8th edition but also recognize its potential pitfalls and limitations.

TNM DESCRIPTORS OF THE NEW 8TH EDITION

The T descriptor of the new 8th edition is composed of tumour size, tumour invasion, and the location of separate tumour with respect to the primary tumor. In the 8th edition the tumor size is more precisely classified in increments of 1 cm. There is a distinct 5 year prognosis for every centimeter increment in tumour size between 1 and 5cm.

New categories of Tis and T1mi were also introduced to clarify the staging of adenocarcinoma. Tis is used for pure lepidic adenocarcinoma which appears as a pure ground glass nodule less than or equal to 3cm. T1mi (minimally invasive ) is used to classify lepidic predominant adenocarcinoma less than or equal to 3cm which appears as part solid nodule with the solid part less than or equal to 0.5cm.

Other changes in the T category include tumour invasion of adjacent structures. Tumour involvement of a main bronchus is downstaged from T3 to T2 regardless of distance from the carina and total atelectasis involving the whole lung is also downstaged from T3 to T2. Diaphragmatic invasion has been upstaged from T3 to T4.

In summary the clinical descriptors in the 8th TNM classification are grouped into five main categories : Tis,T1, T2,T3 and T4.

THE N DESCRIPTOR NO CHANGES

THE M DESCRIPTOR -Change of extrathoracic metastases to M1b (single metastasis ) and M1c multiple metastases in one or several organs)

POTENTIAL PITFALLS AND LIMITATIONS IN IMAGING THE STAGING OF LUNG CANCER.

Lymphangitic carcinomatosis –No descriptor available

Pleural invasion

1. Defined by depth (Pl1,PL2, PL3) requires pathologic examination

2. Visceral pleura (T2)

3. Parietal pleura (T3)

4. Only reliable CT finding of pleural invasion is destruction of bone

5. Pleural puckering, contact with pleura >3cm, obtuse angle with chest wall, and associated pleural thickening are less accurate

Multiple lung lesions

1. Lung cancers with separate tumour nodules

2. Multiple primary lung cancers

3. Multiple ground glass /lepidic lesions

4. Diffuse pneumonic type adenocarcinoma

Lymph node metastases

1. Involvement of distant lymph node groups

2. Number of nodal stations involved vs location alone

CONCLUSION:

There have been major advances in staging lung cancer provided by the 8th edition. Topics still to be addressed include: lymphangitic carcinoma and limitations in the assessment of pleural invasion by imaging.

Reference:

Feng, SH, Yang, Su-Tso: Diagnostic and Interventional Radiology, 2019. Jul. 25: 270-279.

## A11 Risk-based prostate cancer staging – 17:00-17:20

### Petralia Giuseppe (giuseppe.petralia@ieo.it)

#### University of Milan, Italy

We still have a problem with T staging of prostate cancer (PCa). Despite the advances in magnetic resonance imaging (MRI) technology, the reported sensitivity for MRI is only moderate. The largest meta-analysis published, including 9796 patients in 75 studies, reports a sensitivity of only 57% (95% CI: 49-64%) for MRI in detecting extra-prostatic disease. This can be explained with the inability of MRI to detect microscopic extra-prostatic extension (EPE) of the disease. There have been efforts in the last few years to improve the performances of MRI, by developing systems for EPE assessment by MRI. The four main systems (EPE grade, ESUR score, Likert scale and Capsule Contact Length) have been tested a study including 301 patients, with some improvements in the sensitivity values compared to those reported for the MRI in the meta-analysis (about 75% and 57%, respectively), at the expense, however, of the specificity, decreasing from 91% to about 75%. In addition, studies reporting only sensitivity and specificity values are useful to understand the performances of a technique on a given population, but they are of limited utility for assessing single patients. Final users of MRI reports, the urologist and the radiation oncologist, are more interested in negative predictive values (NPV) of MRI for ruling out EPE, as they are more useful for therapy decision making on single patients.

NPV of MRI for ruling out EPE is related to the prevalence of EPE, that is extremely different across the three different risk groups, in which PCa patients are classified by biopsy, digital rectal examination and PSA. For low-risk patients, the reported prevalence of EPE is of 10-15%, while for intermediate-risk and high-risk patients rises up to about 50% and 75%, respectively.

When a radiologist reports an MRI of a low-risk patient, he needs to maximise the specificity in reporting, only reporting for EPE if it is clearly measurable and unequivocal, as the prevalence of EPE is low, so not to deny less invasive approaches to the patient, such as active surveillance, nerve sparing surgery or hypofractionated radiation therapy. For N and M staging, no additional imaging is needed for low-risk-patients.

When a radiologist reports an MRI of an intermediate- or high-risk patient, he needs to maximise the sensitivity in reporting, by mentioning any contact of the tumour with the prostatic capsule, in order to promote appropriate actions from the urologist or radiation oncologist, as the risk of EPE is high (>50%). N and M staging are needed for unfavourable intermediate-risk and high-risk patients.

Next generation imaging (NGI) techniques, including whole-body MRI (WB-MRI), 18F-choline PET/CT and PSMA-PET/CT have shown greater sensitivity and specificity for both N and M staging, compared to conventional imaging, including bone scintigraphy and computed tomography, likely contributing to better patient stratification and therapy decision making. However, it is not known if the management changes induced by NGI have a positive impact on survival. On the other hand, there are studies showing clearly the potential of conventional imaging as predictive and prognostic biomarker in metastatic PCa patients.

## A12 FIGO staging: strength and limitations – 17:20-17:40

### Rosemarie Forstner (r.vliegenthart@umcg.nl)

#### Department of Radiology, University Hospital Salzburg, Paracelsus Medical University, Austria

FIGO classification is the most commonly used staging system by gynaecologists worldwide. It is a dynamic categorization that has undergone several updates driven by evidence based data. Thus, it incorporates new insights in oncology, prognostic features, technical advances and changes in clinical practice (1).

FIGO classification is specific for gynaecologic cancers. Alternatively, the TNM classification can be used, that is applicable for all cancer types. Among British gynaecologists, FIGO tends to be preferred.

FIGO classification is available for all gynaecological malignancies, including ovarian, endometrial, vulvar and cervical cancer, for uterine sarcoma and gestational trophoblastic disease.

The most recent updates of FIGO classification have been published for ovarian cancer in 2014 and for cervical cancer in 2018 (2,3). In staging ovarian cancer, tubal cancer as well as primary peritoneal cancer have been united (2). CT or MRI based staging systems have been adapted to this updated FIGO system and provide pivotal information for treatment planning. However, not only staging alone, but also tumour burden, distribution and patient performance status have to be considered for treatment planning.

The 2018 updated FIGO staging system of cervical cancer has significantly increased the role of imaging for treatment planning (3). New is that clinical staging alone has been replaced by crucial information for treatment planning rendered by either US, CT, MRI or PET/CT. Apart from parametrial invasion, tumour size has become a new major criterion for classification and prognosis. Cut-off values of 2 cm have been introduced in the substages of early cervical cancer. Size has been shown to be associated with the presence of parametrial invasion and of lymph node metastases (3). Exact size and delineation of the tumour is also pivotal in selecting candidates for fertility sparing treatment. The major limitation of FIGO staging of cervical cancer, the lack of integration of lymph nodes, has been overcome by the introduction of stage IIIc. For identification of these lymph nodes PET/CT has been shown to be superior to other imaging techniques. Differentiating between bladder mucosal and bladder wall invasion is justified due to its implications for treatment and prognosis.

As staging information by a single test may be incomplete, the final FIGO stage should incorporate all clinical information and is best assessed in the tumour board consensus.

References

1. Odicino F, Pecoreli S, Ziliani L, Creasman WT. History of the FIGO cancer staging system. Int J Gynaecol Obstet 2008; 101:205-2010

2. Berek SJ Kehoe ST, Kumar L, Frieedlander M. Cancer of the ovary, fallopian tube, and peritoneum. Gynecol and Obstet 2018 https://doi.org/10.1002/ijgo.12614

3. A Kido A, Nakamoto Y. Implications of the new FIGO staging system and the role of imaging in cervical cancer B J Radiol 2021;94: 20201342

## A13 Systemic Staging for Breast Cancer: when and how? – 17:40-18:00

### Sarah Vinnicombe (sarah.vinnicombe@nhs.net)

#### Cheltenham General Hospital, Gloucestershire, UK

Worldwide, breast cancer is the commonest female cancer and the leading cause of female cancer mortality in most countries, with over 2 million new diagnoses and 630,000 deaths in 2018 [1]. Thanks to mammographic screening programmes in most developed countries, many cancers are small at diagnosis. There is a clear relationship between the size of the primary tumour (T stage) and the presence of distant metastases (DM) at presentation. In patients with T1 tumours (smaller than 2cm), the incidence of DM is under 2%. Similarly, if fewer than 4 axillary lymph nodes are involved at imaging, the incidence of DM is under 4%, whereas in patients presenting with T3/4 tumours and N2 nodal disease (four or more abnormal nodes) the incidence of DM rises to 10-20% [2].

Thus, in patients with early stage disease (clinical stage 1-11A), all international guidelines state that screening for asymptomatic DM is not indicated (unless the patient has suspicious symptoms) [3,4]. It frequently generates false positive or indeterminate findings, with significant costs in terms of patient anxiety, resources and radiological follow-up. Though the presence of T3 disease (tumour > 5cm) is often regarded as an indication for whole-body staging, the incidence of DM in clinical stage 11B disease (T3N0, T2N1) is still low with conventional imaging. Units with this policy should robustly audit impact on patient outcomes.

For patients with clinical stage 111 disease, a whole-body technique is indicated for staging. Since the commonest sites of DM are the bones, lungs and liver, contrast-enhanced CT (CECT) is the most commonly used modality, being readily available and having acceptable diagnostic accuracy at these sites. Numerous studies have demonstrated that if the scan volume includes the neck and proximal femora, the additional yield from an isotope bone scan is extremely low [5].

The recent 8th edition of the AJCC TNM manual permits inclusion of tumour immunophenotype, which can be used to modify anatomical TNM staging. Aggressive HER2 amplified and ‘triple negative’ cancers (those without HER2 overamplification, oestrogen or progesterone receptor expression) demonstrate different patterns of metastatic disease, but there is as yet no convincing evidence for altering the strategy for staging according to tumour biology [6]. Again, though many oncologists request systemic staging prior to commencement of neoadjuvant chemotherapy for T1/2 tumours, there is little evidence for the utility of this approach.

More sophisticated cross-sectional techniques including PET-CT and whole body MRI (WB-MRI) are infrequently used for systemic staging in early stage breast cancer. However, there is good evidence that with increasing clinical stage, the incremental diagnostic yield from FDG PET-CT becomes significant [6], particularly for inflammatory breast cancer, where up to 30% of patients may be upstaged [6]. It is also very helpful in the evaluation of equivocal findings at CECT and in the identification of small volume nodal disease. WB-MRI is advantageous in the diagnosis of bone metastases and hepatic metastases, especially in low grade breast cancers such as invasive lobular cancer. With the continued evolution of personalised medicine and targeted therapies, it is likely that the approach to systemic staging will also evolve.

References

1. Bray F, Ferlay J, Soerjomataram I. et al. Global cancer statistics 2018: GLOBOCAN estimates of incidence and mortality worldwide for 36 cancers in 185 countries. CA Cancer J Clin 2018; 68: 394–424

2. Barrett T, Bowden DJ, Greenberg DC, Brown CH, Wishart GC, Britton PD. Radiological staging in breast cancer: which asymptomatic patients to image and how. Br J Cancer 2009; 101: 1522–1528

3. Cardoso F, Kyriakides S, Ohno S, Penault-Llorca F, Poortmans P, Rubio IT et al. Early breast cancer: ESMO Clinical Practice Guidelines for diagnosis, treatment and follow-up. Ann Oncol 2019; 30: 1194–1220

4. Royal College of Radiologists. Guidance on Screening and Symptomatic Breast Imaging (4th edn). https://www.rcr.ac.uk/publication/guidance-screening-and-symptomatic-breast-imaging-fourthedition

5. McCartan DP, Prichard RS, MacDermott RJ, Rothwell RJ, Geraghty J, Evoy D et al. Role of bone scan in addition to CT in patients with breast cancer selected for systemic staging. Br J Surg 2016; 103: 839–844

6. Groheux D, Hindie E. Breast cancer: initial workup and staging with FDG PET/CT. Clin Trans Imaging 2021 Apr 27:1-11 doi: 10.1007/s40336-021-00426-z. Online ahead of print

## A14 How radiotherapy has changed in the last decade – 13:00-13:20

### Julia Murray (Julia.Murray@icr.ac.uk)

#### The Royal Marsden NHS Foundation Trust and Institute of Cancer Research, London

Nearly half of all patients with cancer receive radiotherapy at some point during their cancer treatment. Imaging has an essential role in the planning and delivery of radiotherapy. With the integration of more novel imaging techniques to aid in tumour delineation, these imaging tools have enabled a more personalised approach for target volume identification.

Developments and integration of imaging with radiotherapy technology have enabled image-guided radiotherapy to be standard of care for the delivery of radiotherapy. The tools for image guidance include KV planar imaging, cone beam CT, ultrasound and MRI.

Over the last decade, the rapid technological evolution in radiotherapy delivery, imaging and computing has enabled the increasing use of stereotactic body radiotherapy (SBRT). This is the method of delivering a high dose of external beam radiotherapy very precisely in a small number of fractions. This can now be used in a number of tumour sites for both primary and/or oligometastatic treatments.

Anatomical daily variations do occur during radiotherapy, and over the last decade our ability to adapt to this has increased in sophistication. Adaptive radiotherapy is defined as ‘changing the radiotherapy plan delivered to a patient during a course of radiotherapy to account for temporal changes in anatomy or changes in tumour biology/function’. Examples of adaptive radiotherapy in clinical practice will be discussed. Furthermore, the significant technical advances in radiation treatment planning and delivery have facilitated the re-irradiation of previously exposed volumes.

Additionally, over the last decade, a number of radiotherapy clinical trials have changed clinical practice, not only focussing on optimal dose and fractionation, but trying to determine the biological basis of clinical outcomes.

References:

Jaffray D. Image-guided radiotherapy: from current concept to future perspectives. Nat Rev. Clin. Oncol. 2012: 9: 688-699

Beaton L et al. How rapid advances in imaging are defining the future of precision radiation oncology. BJC 2019: 120: 779-790

Tree AC et al. Stereotactic body radiotherapy for oligometastases. Lancet Oncol 2013: 14: 1: 28-37

Kong V et al. Image-guided adaptive radiotherapy for bladder cancer. Clin Oncol 2021: 33 (6): 350-368

## A15 MR Linac: Radiotherapy of the future – 13:20-13:40

### Alison Tree (Alison.Tree@rmh.nhs.uk)

#### The Royal Marsden NHS Foundation Trust and the Institute of Cancer Research

The MR linac is a ground breaking new radiotherapy machine which combines a 1.5 T MRI scanner with a linear accelerator. The machine was first used in 2018 so clinical experience is in its infancy. However it is clear that this machine promises to create new horizons for cancer therapy.

Radiotherapy is currently guided by CT or implanted fiducials on a daily basis but both of these methods have significant limitations. The MR Linac has three key advantages. Firstly it uses the superior image contrast of MRI to guide radiotherapy, ensuring accurate delivery of treatment. Secondly, cine MRI images are obtained throughout treatment, to ensure no significant movement of the target is occurring. Thirdly the software of the MR Linac also allows the radiotherapy plan to be changed each day, to account for anatomical changes. For example, if the bowel moves close to the prostate, the radiotherapy dose can be reoptimised to avoid the bowel.

There are drawbacks, however. The machine is very resource intensive, currently requiring 4 members of staff to operate at all times. The treatments are relatively slow – for example a prostate treatment can take 45 minutes rather than 10 minutes on a normal machine. Therefore it is important that we set up robust clinical trials to establish whether this extra resource is justified.

This presentation will show some examples of patients who have demonstrably benefited from this new technology and will discuss where this machine may change the way we deliver radiotherapy in the future.

## A16 Targeted therapies in nuclear medicine – 14:00-14:20

### Jolanta Kunikowska (jolanta.kunikowska@wum.edu.pl)

#### Department of Nuclear Medicine, Medical University of Warsaw, Warsaw, Poland

Nuclear medicine is an important tool for cancer management, allows for precise diagnosis on early cancer stage as well, base on it make critical decisions and treatment plan.

Theranostic approach, which means using the same targets for diagnosis and therapy in nuclear medicine is growing. An ideal molecular target for oncological imaging and radionuclide therapy should be specific, easily accessible at the tumour cell plasma membrane, biologically relevant, highly expressed, and not shed into the circulation.

The year, 2021, marked the 80th anniversary of Dr. Saul Hertz first using radioiodine (RAI) to treat a patient with thyroid disease. Iodine uptake by a sodium-iodine pump is the base of RAI application for diagnosis and treatment. Saul Hertz's documented the first experimental data on RAI and apply it in the clinical setting. Nowadays RAI is a commonly use in therapy.

The eighties brought the discovery of somatostatin receptor overexpression on tumour cells, especially on neuroendocrine tumours arising from cells of the endocrine system, with various clinical behaviours. It allows receptor imaging and somatostatin based radiopeptide therapy as a next step. Currently, somatostatin receptor imaging (SRI) is the gold standard in functional imaging for NET and plays a crucial role in the assessment of expression of somatostatin receptors before planned radioisotope treatment.

Initially, therapy trials with [ 111In]In-DTPA-pantetreotide followed by the β-emitters, [90Y]Y-DOTATOC and [177Lu]Lu-DOTATATE, showed a greater impact on tumour volume and survival parameters. The impact of PRRT on overall survival (OS) has been confirmed in a prospective, randomized, phase III trial evaluating the effect of [177Lu]Lu-DOTATATE (NETTER-1).

Finally, in the last years, the prostate-specific membrane antigen (PSMA) base diagnosis and treatment were introduced. Prostate cancer (PCa) is the most common malignancy in men worldwide, leading to substantial morbidity and mortality. The PET/CT imaging with [18F]F/[11C]-Choline has shown limited sensitivity and it is not a target for radionuclide therapy.

PSMA is expressed on the majority of prostate cancer cells, and it represents a cell surface target suitable for imaging metastatic lesions. The first in-vivo study using a urea-based compound targeting PSMA for diagnosis was performed in 2002. From that time several PSMA tracers were tested. [68Ga]Ga- PSMA-11 compound has become of particular interest, because is easy for labelling, stable and allows high contrast imaging by binding to the extracellular domain of PSMA, followed by internalization. ProPSMA report in primary prostate cancer indicates that [68Ga]Ga-PSMA PET/CT is more accurate in the staging of high risk patients than cross-sectional imaging.

The observation of frequent persistent PSMA expression in patients with CRPC has provided the rationale for the recent introduction of PSMA radioligand therapy. The data published in the literature show very promising results of treatment with limited side effects. On 2021 March 23, VISION trial was completed and we are waiting for data analysis.

This is what we have now, but we have in the pipeline lots of new targets. As the radionuclide targeted therapy allows us to maximize treatment effect with minimalize normal cell damage, it is the future of precision medicine.

## A17 Keynote Lecture – 14:50-15:20

### Cancer Survivorship: Imaging implications

#### Robert Huddart

##### Institute of Cancer Research and Royal Marsden Hospital, Sutton Surrey, Uk

Recent years have seen improving outcomes from cancer treatment. In 2019 around 17 million people are estimated to be a cancer survivor in the US and this is projected to increase to over 22million by 2030^1^. Approximately a quarter of these patients are under the age of 60. These patients are at risk of side effects from their cancer care.

Imaging has a positive role in supporting the diagnosis, management, investigation and follow up of patients including the investigation of immediate and long term toxicity of therapy (e.g. cardiotoxicity). The increasing patient workload, intensity of therapy and longevity of patients has fuelled an ever increasing demand for oncology related imaging. At the Royal Marsden the demand for CT imaging has increased by 15% in the last 5 years.

Though difficult to prove, with increasing survivorship in younger patients this increased use of imaging radiation could have adverse consequences. Radiation protection data suggests that a CT abdomen and pelvis exposes an individual to 18 mSV of radiation^2^. This level of exposure is equivalent to 8 years of background radiation associated with a risk of second malignancy of 1 in 2000 per scan. Given many survivors may have in excess of 10 CTs during their cancer journey this becomes a non-trivial risk especially in good prognosis patients.

There is responsibility on oncologists and radiologists to consider these risks. How this may be achieved is discussed in the context of testicular cancer; a highly curable cancer of young men. Clinical trial work on how to achieve equivalent patient outcomes but using less and more targetted imaging; low dose CT and switching to MRI imaging will be discussed.

In conclusion; Oncology related imaging is growing rapidly exposing a growing number of Cancer survivors to significant radiation doses. Oncologists and Radiologists need to ensure that imaging is targeted and is used when it contributes to improved patient outcomes and when used consider the most effective modality.

Sources

1. https://www.cancer.org/content/dam/cancer-org/research/cancer-facts-and-statistics/cancer-treatment-and-survivorship-facts-and-figures/cancer-treatment-and-survivorship-facts-and-figures-2019-2021.pdf].
2. https://www.gov.uk/government/publications/medical-radiation-patient-doses/patient-dose-information-guidance.

## A18 CT/MR LI-RADS: Case-based Workshop – 15:50-16:20

### Jay P. Heiken (heiken.jay@mayo.edu)

#### Mayo Clinic, Rochester, Minnesota, USA

Deaths from liver cancer, primarily hepatocellular carcinoma (HCC), are increasing at the highest rate of all cancer sites. Appropriate choice of treatment depends on accurate diagnosis and staging. Accuracy of classifying liver lesions as HCC or non-HCC on imaging studies is critical to patient management because treatment decisions often are made in the absence of histologic confirmation. LI-RADS is a comprehensive system for standardized interpretation and reporting of CT, MR and ultrasound examinations performed on patients at high risk for HCC (i.e., those with cirrhosis, chronic hepatitis B, or a history of HCC). This workshop will focus on CT/MR LI-RADS.

The CT/MR LI-RADS categories are LR-1 (definitely benign), LR-2 (probably benign), LR-3 (intermediate probability of HCC), LR-4 (probably HCC), LR-5 (definitely HCC), LR-M (probably or definitely malignant, but not specific for HCC), and LR-TIV (tumour in vein). The major imaging features used to diagnose HCC are size, arterial phase hyperenhancement, washout appearance, capsule/pseudocapsule appearance, and threshold growth (size increase ≥ 50% in ≤ 6 months). Only observations that have non-rim arterial phase hyperenhancement and are ≥ 10 mm can be classified as LR-5. In addition to the major imaging features, there are ancillary features favouring malignancy, benignity or HCC in particular. These ancillary features may be used at the radiologist’s discretion to upgrade or downgrade the category by one. Tiebreaking rules should be applied if the radiologist is unsure between two categories. In such a case the category reflecting lower diagnostic certainty should be selected.

When a liver lesion has been treated, the CT/MR LI-RADS treatment response algorithm should be applied. The treatment response categories are LR-TR Nonviable (no lesional enhancement or treatment-specific expected enhancement pattern), LR-TR Equivocal (Enhancement atypical for treatment-specific expected enhancement pattern but not meeting criteria for probably or definitely viable), and LR-TR Viable (nodular, mass-like, or thick irregular tissue in or along the treated lesion, with arterial phase hyperenhancement, washout appearance, or enhancement similar to pretreatment).

## A19 Ablation / irreversible elettroporation – 17:40-18:00

### Mirko D’Onofrio (mirko.donofrio@univr.it)

#### Department of Radiology GB Rossi University Hospital University of Verona Italy

Ablation is frequently used in treating liver tumors in many cases with a complementary role. RFA, among others ablation techniques, is reported to be also a possible palliative treatment inducing intratumoral necrosis and so tumoral cytoreduction. Irreversible Elettroporation is a non thermal technique inducing necrosis and so tumoral cytoreduction. Regarding the guidance for the procedure, ultrasound is fundamental imaging technique. Technical improvements, such as contrast media and fusion imaging, have recently increased precision and safety and reduced procedure-related complications. New treatment US techniques for the ablation of pancreatic tumours, such as contrast-enhanced US and multimodality fusion imaging, have been recently developed and have elicited a growing interest worldwide. Regarding the follow up CT study is always required.

## A20 Session: State of the Art Oncological Imaging: Lymphoma

### WHO classification – implication for imaging – 13:00-13:20

#### Marius E. Mayerhöfer^1, 2^ (mayerhom@mskcc.org)

##### ^1^ Dept. of Radiology, Memorial Sloan Kettering Cancer Center, New York, 10065, USA; ^2^ Dept. of Biomedical Imaging and Image-guided Therapy, Medical University of Vienna, Vienna, 1090, Austria

The current 2016 revision of the WHO classification of tumours of lymphoid tissues recognizes a wide array of different histological lymphoma subtypes [1]. The WHO classification goes far beyond both the classical division into Hodgkin (HL) and Non-Hodgkin lymphomas (NHL), or B-cell and T/NK (natural killer)-cell lymphomas. Since imaging plays a central role for the assessment of lymphomas in both the pre-treatment and the post-treatment setting, radiologists and nuclear medicine physicians need to be aware of the most commonly observed histological lymphoma subtypes, and current recommendations for the use of imaging techniques in these subtypes.

Hodgkin lymphoma (HL), which accounts for <10% of lymphomas and is commonly found in children and young adults. HL is typically involves thoracic and cervical lymph nodes and spreads from one nodal region to the next. Marked cystic components that are frequently mistaken for necrosis are common. Over 60 subtypes of Non-Hodgkin lymphoma exist, the most common ones being diffuse large B-cell lymphoma (DLBCL; 33% of cases), follicular lymphoma (FL; 25% of cases), marginal zone lymphoma (MZL; 10% of cases; subdivided into nodal, splenic, and extranodal/MALT-type MZL), mantle cell lymphoma (MCL; 7% of cases), and small-cell lymphocytic lymphoma/chronic lymphocytic leukemia (SLL/CLL; 7% of cases). While DLBCL is considered an aggressive subtype with a high proliferation rate, FL, MZL, and SLL/CLL are considered indolent, more slowly growing tumour, but with the potential for transformation into aggressive DLBCL. MCL can show an aggressive or an indolent course, or sometimes both in different clones of the same patient.

The Lugano classification of the International Conference on Malignant Lymphoma (ICML) generally recommends two imaging techniques for the work-up of lymphomas: [18F]FDG-PET/CT and (contrast-enhanced) CT [2]. MRI is currently only recommended for assessment of CNS lymphomas, although it may also be a good alternative to CT in young, radiation-sensitive patients and patients with iodine sensitivity. Recent studies have also suggested the use of MRI (including diffusion-weighted MRI) in indolent lymphomas under watchful waiting [3].

[18F]FDG-PET/CT is generally the preferred test for staging and treatment response assessment of the vast majority of lymphomas, including some of the most common subtypes: HL, DLBCL, FL, and MCL [1]. Notably, only PET/CT, but not PET/MRI is currently recommended, possible due to the relative novelty of this hybrid imaging test. For MZL, SLL/CLL, and other entities such as macroglobulinemia Waldenström, [18F]FDG-PET/CT is however not recommended due to their frequently low glucose metabolism – here, CT remains the standard test, despite its limitations for treatment response assessment, in particular its inability to determine between post-treatment fibrosis and viable residual lymphoma. The novel PET radiotracer [68Ga]Pentixafor, which targets the chemokine receptor CXCR4, may possibly represent an option in these lymphoma subtypes [4-6]. With the exception of HL and also DLBCL with focal FDG-avid lesions, no imaging test is currently able to reliably assess bone marrow involvement, especially in indolent lymphomas such as FL, but also in MCL, where bone marrow involvement is found in 55–90% of cases; consequently, bone marrow biopsy remains the standard test [1].

**References**

1. Swerdlow SH, Campo E, Pileri SA, Harris NL, Stein H, Siebert R, Advani R, Ghielmini M, Salles GA, Zelenetz AD, Jaffe ES: **The 2016 revision of the World Health Organization classification of lymphoid neoplasms**. *Blood.* 2016;**127**:2375-2390.

2. Cheson B, Fisher RI, Barrington SF, Cavalli F, Schwartz LH, Zucca E, Lister TA: **Recommendations for initial evaluation, staging, and response assessment of Hodgkin and non-Hodgkin lymphoma: the Lugano classification.** *J Clin Oncol* 2014;**32**:3059-3068.

3. Galia M, Albano D, Tarella C, Patti C, Sconfienza LM, Mulè A, Alongi P, Midiri M, Lagalla R: **Whole body magnetic resonance in indolent lymphomas under watchful waiting: The time is now**. *Eur Radiol* 2018;**28**:1187-1193.

4. Luo Y, Cao X, Pan Q, Li J, Feng J, Li F: ^**68**^**Ga-Pentixafor PET/CT for Imaging of Chemokine Receptor 4 Expression in Waldenström Macroglobulinemia /Lymphoplasmacytic Lymphoma: Comparison to** ^**18**^**F-FDG PET/CT.** *J Nucl Med* 2019;**60**:1724-1729.

5. Haug AR, Leisser A, Wadsak W, Mitterhauser M, Pfaff S, Kropf S, Wester HJ, Hacker M, Hartenbach M, Kiesewetter-Wiederkehr B, Raderer M, Mayerhoefer ME: **Prospective non-invasive evaluation of CXCR4 expression for the diagnosis of MALT lymphoma using [**^**68**^**Ga]Ga-Pentixafor-PET/MRI**. *Theranostics* 2019;**9**:3653-3658.

6. Mayerhoefer ME, Haug A, Jäger U, Pichler V, Pfaff S, Wester HJ, Hacker M, Kazianka L, Staber PB: **In Human Visualization of Ibrutinib-Induced CLL Compartment Shift**. *Cancer Immunol Res*. 2020;**8**:984-989.

## A21 Extranodal manifestations of lymphoma in the abdomen – 14:00-14:20

### Isaac R Francis (ifrancis@umich.edu)

#### Department of Radiology, Michigan Medicine, University of Michigan, Ann Arbor, Michigan, 48109, USA

**Introduction:**

The WHO based classification is based on the cell of origin as to B or T cell or NK (natural killer) cell

In the United States, Non-Hodgkin lymphoma (NHL) is more common than Hodgkin lymphoma/disease (HD) and over 50% of NHL are either the aggressive diffuse large B-cell variant (DLBCL) or the indolent follicular type (FL) [1]

By definition extra nodal lymphoma denotes involvement of sites other than lymph nodes, thymus, tonsils, pharyngeal lymphatic ring (Ring of Waldeyer), and the spleen except in HD. Extra nodal involvement is more common in NHL, and AIDS-related and Post-Transplant Lymphoproliferative Disorder [PTLD]are more often extra nodal [2,3].

Primary lymphoma denotes involvement of an organ and adjacent lymph nodes and secondary, involvement of more than one extra nodal site or involvement of lymph nodes other than those adjacent to an organ; the latter being more common than primary involvement [2,3].

In decreasing order of frequency sites of abdominal involvement are: the spleen, GI tract, pancreas, abdominal wall, GU tract, adrenal glands, peritoneal cavity, and the biliary tract.

**Spleen:**

Can be involved in 20-30% of cases, organomegaly diffuse infiltration and focal lesions are the patterns of involvement and typically the lesions are more homogenous and hypovascular than metastases, and larger than fungal abscesses [2,4].

**Liver:**

Less commonly involved than spleen; 10-15%. Primary involvement is rare and seen as dominant focal mass. Secondary involvement usually mutifocal masses with associated splenic and nodal involvement [2,5].

**Pancreas:**

Can present as focal mass or masses mimicking pancreatic adenocarcinoma, but vascular encasement and pancreatic duct obstruction are rare: or diffuse infiltration mimicking pancreatitis [2,6].

**Renal:**

Usually secondary to direct spread from contiguous sites of disease or haematogenous. Multifocal masses are most common manifestation, unifocal masses, spread from retroperitoneum mimicking central hilar mass, and perirenal involvement are lesser common manifestations. Nephromegaly due to diffuse infiltration is uncommon [7,8].

**Adrenal:**

Rare; with primary involvement seen as large bilateral masses in 50% of cases: can also manifest as thickened and enlarged adrenal glands [8,9].

**Gastrointestinal Tract:**

Stomach most common site, with small bowel, colon, and oesophagus in decreasing order. Can have nodular, infiltrative, cavitary, polypoid or ulcerative and aneurysmal appearance. In contrast to adenocarcinoma, gastric lymphoma maintains distensibility and preservation of perigastric fat planes. Colorectal lymphoma may be associated with HIV, PTLD and IBD [2].

**Peritoneum:**

Very uncommon, mimics abdominal carcinomatosis [2].

**References:**

1. Matasar MJ and Zelenetz AD. Overview of lymphoma diagnosis and management. Radiol Clin N Am 2008;46:175-198

2. Leite, NP et al. Cross-sectional Imaging of extra nodal involvement in abdomino-pelvic lymphoproliferative malignancies. Radiographics 2007;27:1613-1634

3. Li M et al. Imaging findings of primary splenic lymphoma: A review of 17 cases in which diagnosis was made at splenectomy. PLOS ONE; 2013;8: Issue 11.e80264.

4. Rajesh S et al. The imaging conundrum of hepatic lymphoma revisited. Insights Imaging 2015;6:679-692

5. Anand D et al. Current update on primary pancreatic lymphoma. Abdom Radiol 2016;41:347-355

6. Ganeshan D et al. Imaging of primary and secondary renal lymphoma. AJR 2013; 201:W712-W719

7. Rohena-Quinquilla IR et al. Imaging of extra nodal genitourinary lymphoma. Radiol Clin N AM 2016; 54:747-764

8. Zhou L et al. Primary adrenal lymphoma: radiologic; pathological, clinical correlation. Eur J Radiol 2012;81:401-405

9. Gollub, M. Imaging of Gastrointestinal lymphoma. Radiol Clin N Am 2008;46:287-312

## A22 Oncological Imaging: Practical approach – Mimics of malignancy: pancreas – 15:30-15:50

### Maria Antonietta Bali (mbali@ulb.ac.be)

#### Department of Radiology, Institut Jules Bordet, ULB, Brussels, Belgium

There are several benign conditions that may mimic pancreatic adenocarcinoma (PDAC) at non-invasive imaging making the differential diagnosis very challenging. By integrating imaging findings with clinical (patient medical history and symptoms), laboratory and pathologic data a correct diagnosis may be achieved so to avoid unnecessary pancreatic surgical resection associated to non-negligible morbidity and mortality, even in high volume centres.

Focal pancreatic lesion mimicking PDAC often appear as hypodense/hypovascular lesion at contrast-enhanced CT. To increase the diagnostic performance, MR with diffusion weighted imaging (DWI) and MRCP sequences should be performed and will provide insights of lesion tissue characteristics and of pancreatic and biliary ducts.

The most common conditions that may mimic PDAC are mass-forming pancreatitis, paraduodenal and autoimmune pancreatitis (AIP). These benign inflammatory conditions share with PDAC similar intra-lesional histopathologic characteristics such as extensive fibrotic changes and poor vascular density. These histopathologic hallmarks may explain the overlapping imaging features observed on both conventional and functional imaging and are responsible for the diagnostic challenge. DWI has been increasingly used in abdominal imaging and more particularly to differentiate AIP from PDAC: lower ADC have been reported in AIP compared to PDAC and several optimal cut-off values have been suggested to discriminate the two entities. More recently to differentiate between AIP and PDAC, radiomics-based models obtained on conventional CT images have been suggested which reported high sensitivity and specificity in predicting both conditions. These results are very encouraging, however the complexity of the image analysis procedure makes this approach available only in an academic environment and does not yet allow its application in a clinical routine setting.

Of worthy value for the differential diagnosis between these benign inflammatory conditions and PDAC are the presence at imaging of secondary signs such as ‘duct penetrating sign”, skip strictures of the CBD or/and the MPD, the presence of capsule-like rim, intra-lesion calcifications distribution, lesion-to-vessel contact, delayed lesion enhancement. Moreover, to differentiate AIP from PDAC the presence of extra-pancreatic manifestations and the favourable response to steroids after two-week test are important diagnostic landmark.

Besides inflammatory lesions, other less current benign conditions can occur within the pancreas, which are responsible for diagnostic challenges. For example, the presence of intra-pancreatic fat infiltration often localized in the pancreatic head may mimic a focal lesion suggesting the diagnosis of PDAC based on its hypodense/hypovascular behavior on conventional CT. The lack of associated secondary imaging findings and a complementary imaging study with MR fat saturated sequences will allow the correct diagnosis and avoid further investigation or unnecessary surgical intervention.

Serous microcystic cystadenoma depicted on conventional CT in some cases may be misdiagnosed as a PDAC lesion because of its “pseudosolid” hypodense appearance demonstrating hypovascular behaviour. A complementary MR study with T2 weighted and MRCP sequences will provide the right diagnosis and avoid other costly and more invasive investigations.

## A23 Oncological Imaging: Practical Approach - Mimics of Malignancy: Adnexa – 15:50-16:10

### Gabriele Masselli (gabriele.masselli@uniroma1.it)

#### Radiology Department, Umberto I Hospital Sapienza University, Rome, Italy

The evaluation of pelvic mass presents a diagnostic challenge since it can be difficult to assess their origin and the overlap in imaging features.

Imaging modalities are required for the detection and characterization of the pelvic lesions distinguishing tumours from their mimics, that is critical for the correct clinical and surgical management.

When evaluating a pelvic mass the first step is to identify both ovaries.

Normally, ovaries are almond-shaped, and in pre-menopausal women demonstrate multiple small follicles located preferentially in the periphery of an inhomogeneous stroma.

The capsule may be evident as a dark line surrounding the well-defined follicles.

In the post-menopausal women the involuted and atrophic ovaries contain fewer, smaller or no cyst and may become difficult to identify.

In clinical routine, 5–25 % of adnexal lesions remain indeterminate after ultrasound (US) [1]. Even using the International Ovarian Tumour Analysis group (IOTA) simple rules, 22 % of lesions remained indeterminate on US [1-2]. Most of these are common benign entities including haemorrhagic lesions, fat-poor mature teratomas, uterine leiomyomas and ovarian fibromas. The clinical impact of defining whether an indeterminate mass is benign or malignant is crucial to select the patients, that should undergo surgery.

Women with suspected ovarian cancer may require radical surgery whereas benign adnexal masses may either be managed conservatively.

MRI studies are required to evaluate indeterminate lesions at US [3].

O-RADS MRI was created to standardize descriptions of ovarian and adnexal pathology, allowing a better risk stratification and addressing a significant clinical issue [4].

Using this MR score in clinical practice for masses that are indeterminate at US, a tailored, patient-centered approach is provided, preventing unnecessary surgery, less extensive surgery, or fertility preservation.

In conclusion MRI is a useful complementary imaging technique for assessing sonographically indeterminate masses. MRI categorization allows confident diagnosis in the majority of adnexal masses and in sonographically indeterminate masses, complementary MRI assists in triaging patient management.

References

1. Froyman W, Landolfo C, De Cock B, et al. . Risk of complications in patients with conservatively managed ovarian tumours (IOTA5): a 2-year interim analysis of a multicentre, prospective, cohort study. Lancet Oncol. 2019;20(3):448-458

2. Timmerman D, Van Calster B, Testa A, et al. . Predicting the risk of malignancy in adnexal masses based on the Simple Rules from the International Ovarian Tumor Analysis group. Am J Obstet Gynecol. 2016;214(4):424-43

3. Forstner R, Thomassin-Naggara I, Cunha TM, et al ESUR recommendations for MR imaging of the sonographically indeterminate adnexal mass: an update. Eur Radiol. 2017 ;27(6):2248-2257.

4. Thomassin-Naggara I, Poncelet E, Jalaguier-Coudray A et al. Ovarian-Adnexal Reporting Data System Magnetic Resonance Imaging (O-RADS MRI) Score for Risk Stratification of Sonographically Indeterminate Adnexal Masses. JAMA Netw Open. 2020;3(1):e1919896.

## A24 Oncological Imaging: Practical Approach - Paediatric oncological emergencies – 16:40-17:00

### Kieran McHugh (kieran.mchugh@gosh.nhs.uk)

#### Great Ormond Street Hospital, London, UK

Although much rarer than adult cancer some paediatric malignancies do also present as an emergency. Conventional radiography can be helpful in suspected bowel perforation, bowel obstruction or airway obstruction from mediastinal masses; however, ultrasound and CT are frequently more informative in general. MR imaging, whilst excellent for characterising and staging a primary malignancy, has less of a role in the emergency scenario apart from in neurological emergencies, such as suspected spinal cord compression. The other major neurological emergency, which can be caused by a variety of brain tumours, is that of raised intracranial pressure, the acute management of which can be managed by CT scanning in the first instance. Given that imaging plays a pivotal role in these and other emergency scenarios, the radiologist is in a privileged position whereby a high level of interpretative acumen can make the difference between life and death or serious long-term disability. Other non-neurological emergencies include diaphragm splinting from enormous abdominal or hepatic masses such as neuroblastoma, pericardial tamponade, massive pleural effusions, SVC obstruction, urinary tract obstruction and abdominal compression syndrome. Venous thromboembolism is also a recognised problem at presentation in Wilms’ tumours in particular and occasionally also with hepatic tumours. Examples of all of these problems will be presented with advice on a practical approach to management. It is noteworthy that most children suffering from an acute complication may not initially present to a specialist centre, thus it is the responsibility for all general radiologists to have a basic awareness of such cases.

Reference:

Imaging in Pediatric Oncology. Editors. Stephan D. Voss, Kieran McHugh. Springer 2019. ISBN 978-3-030-03776-5 ISBN 978-3-030-03777-2 (eBook) 10.1007/978-3-030-03777-2

## A25 Brain haemorrhage/drug induced encephalopathy – 17:20-17:20

### Giovanni Morana (giovanni.morana@unito.it)

#### Department of Neurosciences, University of Turin, TO, 10126, Italy

Brain haemorrhage is a common Central Nervous System (CNS) complication which accounts for about 50 percent of the cerebrovascular accidents in oncological patients [1,2]. It may result from direct or indirect effects of cancer, or from systemic effects of oncological therapy during treatment and/or post-remission [1-3].

Drug induced encephalopathy can be determined by a wide and heterogeneous group of medications and the two most common CNS injury patterns are represented by Posterior Reversible Encephalopathy Syndrome (“peripheral pattern”) and Acute Toxic Leukoencephalopathy (“central pattern”) [4,5]. Different patterns of injury can be caused by several drugs, and may involve deep structures such as basal ganglia or brainstem (“deep pattern”) [5].

Early detection is extremely important to suspend the offending medication and to provide early treatment of neurotoxic side effects [6,7]. In order to appropriately interpret CNS imaging findings of patients experiencing brain haemorrhage/drug induced encephalopathy an accurate knowledge of the underlying disease, treatment plan, and timing of onset and duration of symptoms is required [1,8].

In oncological patients with a suspected brain haemorrhage Computed Tomography still remains the first-line imaging technique due to its speed, widespread availability, and high sensitivity/specificity for acute bleeding [1]. However, Magnetic Resonance Imaging (MRI) represents the gold standard imaging technique to better characterize haemorrhagic lesions and for the assessment of drug induced neurotoxicity. MRI also allow to differentiate treatment-related toxicity from other conditions such as progressive underlying disease, infectious or paraneoplastic neurologic disorders [9].

The aim of this work will be to provide a review of the most common neuroimaging patterns of brain haemorrhage/drug induced encephalopathy in oncological patients.

References

1. Graus F, Rogers LR, Posner JB. Cerebrovascular complications in patients with cancer. Medicine. 1985;64:16-35.

2. Velander AJ, DeAngelis LM, Navi BB. Intracranial hemorrhage in patients with cancer. Curr Atheroscler Rep. 2012;14:373-81

3. Navi BB, Reichman JS, Berlin D, Reiner AS, Panageas KS, Segal AZ, DeAngelis LM. Intracerebral and subarachnoid hemorrhage in patients with cancer. Neurology. 2010;74:494-501.

4. Dietrich J, Klein JP. Imaging of cancer therapy-induced central nervous system toxicity. Neurol Clin. 2014;32:147-57.

5. Iyer RS, Chaturvedi A, Pruthi S, Khanna PC, Ishak GE. Medication neurotoxicity in children. Pediatr Radiol. 2011;41:1455-64.

6. Rossi Espagnet MC, Pasquini L, Napolitano A, Cacchione A, Mastronuzzi A, Caruso R, Tomà P, Longo D. Magnetic resonance imaging patterns of treatment-related toxicity in the pediatric brain: an update and review of the literature. Pediatr Radiol. 2017;47:633-648.

7. Rossi A, Morana G, Gandolfo C, Severino M. Neuroradiology of chemotherapeutic. Neurotoxicity in children. Neuroradiol J. 2010;23:183-90.

8. Stone JB, DeAngelis LM. Cancer-treatment-induced neurotoxicity--focus on newer treatments. Nat Rev Clin Oncol. 2016;13:92-105.

9. Albano D, Benenati M, Bruno A, Bruno F, Calandri M, Caruso D, et al. Imaging side effects and complications of chemotherapy and radiation therapy: a pictorial review from head to toe. Insights Imaging. 2021;12:76.

## A26 Staging of uterine malignancies and the impact on treatment selection and planning – 17:40-18:00

Evis Sala (es220@cam.ac.uk)

Magnetic resonance imaging (MRI) plays a crucial role in treatment selection and planning in patients with endometrial and cervical cancer (CC).

Endometrial Cancer:

MRI is the imaging modality of choice for assessment of patients with EC. It allows better risk assessment and ultimately guides the surgical planning. The combination of conventional T2-weighted sequence (T2WI) with diffusion (DWI) and dynamic contrast enhanced (DCE) MRI provides the “one-stop shop” approach for the evaluation of the extent of disease in patients with EC. High-resolution axial-oblique T2WI perpendicular to the endometrium are essential for accurate evaluation of depth of myometrial invasion. A slice thickness of 4mm and the use of non-fat suppressed sequences is recommended. DWI images should be obtained with a minimum of two b values of 0 and 800-1000 s/mm2 in the same orientation as the sagittal and axial oblique T2WI. DCE-MRI can be used as an adjunct particularly in those patients with total hip replacement when metal artefact limits the value of DWI. MRI staging protocol for EC patients should also include a large-field-of-view axial T1WI and/or T2WI images of the abdomen and pelvis in order to identify enlarged lymph nodes, hydronephrosis and bone marrow changes.

MRI has a reported accuracy of 85-93% in delineating the extent of the EC and is the imaging modality of choice to determine the depth of myometrial invasion preoperatively. The latter is the most important morphologic prognostic factor, correlating with tumour grade, presence of lymph node metastases and overall survival. MRI can confirm the absence of myometrial and cervical stroma invasion, ovarian metastases and the absence of lymphadenopathy, all eligibility criteria for fertility sparing treatment in patients with grade 1 EC who desire fertility preservation.

Cervical Cancer:

MRI is the single best modality for evaluation of tumour extent in patients with cervical cancer enabling accurate treatment selection and planning. The MRI protocol for CC is similar to EC, the only difference is that the high-resolution axial-oblique T2WI and DWI are obtained perpendicular to the cervix and are essential for accurate detection of parametrial invasion (stage IIB).

The 2009 FIGO staging for cervical cancer recommended the use of imaging modalities including MRI if available. The revised 2018 FIGO staging incorporated imaging findings for evaluation of tumour size, tumour extent and the assessment of lymph node metastasis. A new cut-off value of 2cm was added to the previous 4cm cut-off for stage IB tumours due to the difference in prognosis and increased use of fertility sparing surgery. Tumour measurements on MR imaging shows excellent correlation with pathological size. The addition of DWI to T2WI has led to improvement in the accuracy of assessment of parametrial invasion, which in turn enables patient triaging to surgery versus external beam radiotherapy (if parametrial invasion is present).

Regarding lymph node metastasis, PET/CT and more recently PET/MRI shows superior diagnostic accuracy compared with MRI and CT alone. A combination of MRI and PET/CT is also recommended for surveillance of recurrent disease.

References:

1. Balleyguier C, Sala E, Da Cunha T et al.. Staging of uterine cervical cancer with MRI: guidelines of the European Society of Urogenital Radiology. Eur Radiol 2011;21(5):1102–1110.

2. Nougaret S, Horta M, Sala E, Lakhman Y, Thomassin-Naggara I, Kido A, Masselli G, Bharwani N, Sadowski E, Ertmer A, Otero-Garcia M, Kubik-Huch RA, Cunha TM, Rockall A, Forstner R. Endometrial Cancer MRI staging: Updated Guidelines of the European Society of Urogenital Radiology. Eur Radiol. 2019 Feb;29(2):792-805.

3. Sala E, Rockall AG, Freeman SJ, Mitchell DG, Reinhold C. The added role of MR imaging in treatment stratification of patients with gynecologic malignancies: what the radiologist needs to know. Radiology. 2013 Mar;266(3):717-40

4. Freeman SJ, Aly AM, Kataoka MY, Addley HC, Reinhold C, Sala E. The revised FIGO staging system for uterine malignancies: implications for MR imaging. Radiographics. 2012 Oct;32(6):1805-27.

## A27 Residents’ Corner: On-demand stream

### Staging Head & Neck Cancer

#### Ann D King (king2015@cuhk.edu.h)

##### Chinese University of Hong Kong

Introduction

Head and neck cancers are staged according to the UICC and AJCC systems, currently the 8th editions, for primary tumour (T stage), cervical nodal metastases (N stage) and distant metastases (M stage). A wide range of different cancer types and cancer sites are found in the head and neck which is reflected in the many different staging systems applied to this region. This lecture will focus on staging squamous cell carcinoma at sites in the pharynx (nasopharynx, oropharynx and hypopharynx), and sites that lie anterior to the pharynx (sinonasal, oral cavity and larynx, respectively). General principles that cover staging will be demonstrated and modalities for imaging discussed.

T Stage

In general, the T staging of local spread (T1-4) progresses from superficial involvement of the site/subsites, to spread to adjacent sites and then to deep invasion into fat planes & muscle; bone & cartilage; orbit, brain & cranial nerves; subcutaneous soft tissues, skin, prevertebral space, carotid sheath & mediastinum. However, there are important variations, so each site has its own staging system. Tumour size also contributes to the T1-T3 stage of oropharyngeal, hypopharyngeal and oral cavity cancers (using 2 and 4 cm thresholds for maximum overall size with the addition of 5 and 10 mm for maximum depth of oral cavity tumours). Cases will be used to illustrate assessment of these features for staging and treatment planning.

N Stage

Nodal diagnosis by MRI/CT/ultrasound depends on imaging criteria such as size (measured in the minimum axial dimension), necrosis and extranodal spread, with the addition of peripheral vascularity and loss of the hilum on ultrasound and FDG activity on PET. There is also a higher index of suspicion for borderline nodes when they are isolated to nodal groups in the expected course of nodal spread.

Nodal staging (N0-N3) depends on size (measured in the maximum dimension using 3 cm and 6 cm thresholds); number (one node vs multiple nodes) and side (unilateral vs contralateral/ bilateral). Currently for staging purposes, extranodal spread is assessed clinically but it is still important to comment on extension into adjacent structures as well as the relationship of nodes to major vessels. Nodal staging is similar for all sites with the exception of P16 positive oropharyngeal carcinoma and nasopharyngeal carcinoma (virus-related cancers) for which there are minor adjustments, including addition of the lower limit of nasopharyngeal carcinoma nodal metastases (above vs below the lower border of the cricoid cartilage). Cases will be used to illustrate assessment of these features for staging and treatment planning.

Distant Metastases Stage (M0 and M1) and Second Primary Tumours

Distant metastases are uncommon at initial presentation, but head and neck cancers are associated with second primary tumours which tend to arise in the head and neck or lung.

Structured reporting

Head and neck cancers are complex cancers to stage. A structured report based on the TNM staging provides a valuable basic template for each cancer site. However, staging systems do not provide all the information that is required for clinical management and so each basic template needs to be adapted for each cancer site.

## A28 Artificial intelligence in gynaecology oncology imaging

### Gigin Lin (giginlin@cgmh.org.tw)

#### Department of Medical Imaging and Intervention, Chang Gung Memorial Hospital at Linkou and Chang Gung University, Taoyuan, Taiwan

Artificial intelligence (AI) is expected to significantly influence the practice of medicine, and the delivery of healthcare in the near future. It is critical that radiologists understand the basic technology aspects of artificial intelligence, so we can see beyond the hype, and acknowledge the limitations and opportunities. This presentation aims to serve as a short and digestible repository of information, and details every radiologist might need to know in the age of AI. We will describe the basic of machine learning and radiomics, and update their applications in gynecology oncology. My talk will cover the following topics: Where are we now in 2021? Machine learning, deep learning and radiomics. Then we will break down to check the progress in cervical cancer, ovarian cancer, endometrial cancer, and uterine sarcoma. There are ongoing researches of machine learning and deep learning in Gynecology Oncology Imaging. The major progress is in cervical cancer, ranging from tumour characterization, tumour delineation, for radiation planning, surgical planning, and survival prediction. Minor progress also shown in ovarian cancer, with emphasis on recurrence or survival prediction. Some progress is starting in endometrial cancer, to determine the myometrial invasion depth and lymph node status. Very few progresses in uterine sarcoma, possibly due to the limitation of data. Overall, artificial intelligence in Gynecology Oncology Imaging is not yet a reality, but we can expect how to implement those algorithms in clinical environment will be the next big thing. We hope this talk will inspire the audience to think about the potential benefits, dangers, challenges of AI, as well as attempt to provide a futuristic vision to use it in an everyday medical practice.

References

1. Liu Y, Chen PC, Krause J. How to Read Articles That Use Machine Learning: Users' Guides to the Medical Literature. JAMA. 2019:12;322(18):1806-1816.

2. Meskó B, Görög M. A short guide for medical professionals in the era of artificial intelligence. NPJ Digit Med. 2020:24;3:126

3. Lin YC, Lin CH, Lin G. Deep learning for fully automated tumor segmentation and extraction of magnetic resonance radiomics features in cervical cancer. Eur Radiol. 2020:30(3):1297-1305.

4. Yang LY, Siow TY, Lin G. Computer-Aided Segmentation and Machine Learning of Integrated Clinical and Diffusion-Weighted Imaging Parameters for Predicting Lymph Node Metastasis in Endometrial Cancer. Cancers. 2021:19;13(6):1406

5. Benjamens S, Dhunnoo P, Meskó B. The state of artificial intelligence-based FDA-approved medical devices and algorithms: an online database. NPJ Digit Med. 2020: 11;3:118.

## A29 Open access labelled data to advance machine learning research in cancer imaging

### Fred Prior (FWPrior@uams.edu)

#### Departments of Biomedical Informatics and Radiology, University of Arkansas for Medical Sciences, Little Rock, Arkansas, USA

The COVID-19 pandemic has highlighted the need to rapidly acquire and globally share data to support research, clinical decision making and policy setting[1]. Nowhere is this more important than in the advancement of AI applications in healthcare[2]. For AI algorithms to become clinically useful they must be trained on data that appropriately represents the variance in the human population, the presentation of disease and the data collection systems[3]. While distributed ML techniques[4, 5] make it possible for the data to remain private, open access data and open science facilitate collaboration and rapid advancement[6].

Cancer imaging has seen rapid advancements in the areas of quantitative image analyses (radiomics) and the application of machine learning, particularly deep learning techniques[7]. The bulk of this work has been based on supervised learning techniques which require labeled data for training, testing and validation. While some researchers have focused on techniques that can draw insights from limited samples[8], most techniques can only generalize when trained on large, representative samples of labeled data.

What is labeled data? The answer to this question depends on the research question being asked. It can be as simple as a binary outcome (cancer/no cancer) that can be used to partition the training data. Conversely it can be a complex combination of clinical parameters, pathology results and image annotations, or even the results of a previous radiomics or segmentation analysis. This complexity makes the acquisition and sharing of labeled data difficult.

The production of labeled data most frequently relies on the effort of human experts to annotate images with boundaries and measurements. This is a time consuming and expensive process which limits the quantity of such data. Advanced techniques such as data augmentation, synthetic data and transfer learning have been used to extend human labeled data, but the gold standard continues to be human labeling[3].

Because labeled data is both complex and expensive to produce, the need to openly share such data is obvious. In cancer imaging the number of resources sharing labeled data is quite limited and they are not consistent in the way labeled data is managed. While adherence to the FAIR principles[9] is now common practice for images, associated labels are not always easily found or accessed. Mechanisms for identifying labeled data across repositories are largely non-existent. These limitations severely constrain our ability to create truly generalizable AI applications.

By focusing on the unique issues related to labeled data we are working to enhance open access research data repositories to collect, curate, and provide access to ever larger volumes of such data. There is an urgent need for international collaborations to create the truly representative, large scale data cohorts needed to train generalizable AI applications.

References

1. Park JJ, Mogg R, Smith GE, Nakimuli-Mpungu E, Jehan F, Rayner CR, et al. How COVID-19 has fundamentally changed clinical research in global health. The Lancet Global Health. 2021;9(5):e711-e20.

2. Davenport T, Kalakota R. The potential for artificial intelligence in healthcare. Future Healthc J. 2019;6(2):94-8.

3. Prior F, Almeida J, Kathiravelu P, Kurc T, Smith K, Fitzgerald T, et al. Open access image repositories: high-quality data to enable machine learning research. Clinical radiology. 2019.

4. Reina GA, Gruzdev A, Foley P, Perepelkina O, Sharma M, Davidyuk I, et al. OpenFL: An open-source framework for Federated Learning. arXiv preprint arXiv:210506413. 2021.

5. Chang K, Balachandar N, Lam C, Yi D, Brown J, Beers A, et al. Distributed deep learning networks among institutions for medical imaging. Journal of the American Medical Informatics Association. 2018;25(8):945-54.

6. Pascu C, Szkuta K, Von Schomberg R, Karalopoulos A, Repanas K, Schouppe M. Open Science, Open Data and Open Scholarship: European Policies to Make Science Fit for the 21th Century. 2020.

7. Napel S, Mu W, Jardim‐Perassi BV, Aerts HJ, Gillies RJ. Quantitative imaging of cancer in the postgenomic era: Radio (geno) mics, deep learning, and habitats. Cancer. 2018;124(24):4633-49.

8. Kim S, Dougherty ER, Barrera J, Chen Y, Bittner ML, Trent JM. Strong feature sets from small samples. Journal of Computational biology. 2002;9(1):127-46.

9. Wilkinson MD, Dumontier M, Aalbersberg IJ, Appleton G, Axton M, Baak A, et al. The FAIR Guiding Principles for scientific data management and stewardship. Scientific data. 2016;3.

## O1 Relative Fat Fraction of malignant bone lesions shows good interobserver agreement and aids disease detection

### F. Castagnoli, R. Donners, N. Tunariu, K. Downey, C. Messiou, D. Kow

#### Royal Marsden Hospital, Sutton, London

##### **Correspondence:** F. Castagnoli (francy-cast@hotmail.it)

Aim: To compare the relative fat fraction (rFF) of active bone lesions from breast, prostate and myeloma malignancies and normal bone marrow; to assess its inter-reader agreement.

Materials and Methods: Patients with myeloma (n=32), breast (n=26) and prostate cancer (n=52) were retrospectively evaluated. 109 baseline axial rFF maps from whole-body MRI (including Dixon T1w and DWI from skull base to mid-thigh) were reviewed by two radiologists. The rFF maps were calculated by (fat-only image) / (fat-only image+water-only image)x100%. Regions of interest for up to four focal active bone lesions were drawn on rFF maps, one each at the cervicothoracic spine, lumbosacral spine, pelvis and extremity. The mean and standard deviation of rFF(%) were recorded. The rFF of normal marrow was measured in patients without diffuse pelvic disease (n=88). We compared the rFF of bone lesions and normal marrow using Mann-Whitney test. Interobserver agreement was assessed by interclass correlation.

Results: Malignant bone lesions showed significantly lower median rFF (13.87%) compared with normal bone marrow (89.76%) with little overlap (P<0.0001). There was no significant difference in the median rFF of malignant bone lesions from myeloma (13.12%), breast (14.46%) and prostate cancer (13.67%) (p>0.017, Bonferroni correction) and in the median rFF of bone disease according to their anatomical locations (p>0.008, Bonferroni correction). There was excellent interobserver agreement of rFF measurements (0.976). Conclusion: The low rFF of active bone lesions provides high image contrast relative to normal marrow that can enhance disease detection. The rFF has excellent inter-reader agreement, which is useful for assessing treatment response.

## O2 Borderline Ovarian Tumours- What the Radiologist needs to know

### Renn A, Kafaei L, Downey K, Gagliardi, Butler J, Barton D, Ind T, Nobbenhuis M, Sohaib A

#### The Royal Marsden Hospital, Sutton, UK

##### **Correspondence:** Renn A (arenn55@gmail.com)

Learning Objectives

1. Understand the pathological classification of ovarian tumours in particular borderline tumour

2. Recognise the imaging features that favour borderline ovarian tumour

3. Identify the features concerning for malignancy

4. How imaging affects management decision

Content Organisation

1. **Introduction**: Brief discussion regarding the epidemiology and pathology of Borderline Ovarian Tumours (BOTs)

2. **Detection and Characterisation**: to include the benefits and limitations of ultrasound, CT and MRI. The recommended imaging protocol for optimal assessment.

3. **Imaging features of epithelial subtypes of Borderline Ovarian Tumours, with pictorial demonstration** (serous, mucinous, endometroid, clear cell and Brenner): To include size, uni/bilateral examples and typical imaging

4. **The features which raise the concern of malignancy**: including clinical history, imaging findings and biochemical markers

5**. Staging and treatment planning, management decision:** succinct discussion regarding details of current FIGO guidelines, treatment planning*,* lymphadenectomy, fertility and cystectomy versus oophorectomy

Conclusion:

The diagnosis of Borderline Ovarian Tumours is frequently challenging for the radiologist. However, it is important for the radiologist to be familiar with their imaging appearances, as patients are typically younger and may benefit from alternative, less aggressive and fertility-sparing approaches.

## O3 Optimisation of acquisition parameters for whole-body diffusion weighted MRI in patients with metastatic melanoma

### A Knill, MD Blackledge, A Curcean, J Larkin, S Turajlic, DM Koh, C Messiou, JM Winfield

#### The Institute of Cancer Research, London. The Royal Marsden NHS Foundation Trust, Sutton, London

##### **Correspondence:** A Knill (annemarie.knill@icr.ac.uk)

Aim: To measure optimised b-values for acquisition of whole-body diffusion-weighted MRI (WB-MRI) by applying a model to apparent diffusion coefficient (ADC) values in a retrospective cohort of patients with metastatic melanoma.

Methods: ADC was calculated in 125 lesions in a retrospective cohort (14 examinations from 11 patients). The optimum b-value for the acquisition of diffusion-weighted imaging (DWI) was estimated by applying a model relating the theoretical error in ADC measurements to the distribution of population ADC estimates from the clinical data. Phantom experiments were used to assess the effect of b-values on distortion and minimum echo time (TE).

Results: The mean age of patients was 58 years (range 22–73 years) and the most common location of metastases was bone (48), followed by liver (47). Mean ADC of all lesions was 1.18 (10th and 90th percentile: 0.72–1.81)x10-3 mm2/s and was lowest in presacral nodules: 0.67 (0.48–0.86)x10-3 mm2/s, and highest in intramuscular deposits: 1.49 (1.20–1.77)x10-3 mm2/s. The optimum b-value for ADC calculation was predicted as 1100 (10th and 90th percentile: 740–1790) s/mm2 considering all lesions together. Geometric distortion and minimum TE increased with increasing b-value.

Conclusions: Using a theoretical model, a b-value of 1100 (10th and 90th percentile: 740–1790) s/mm2 is optimal for ADC calculation in patients with melanoma. However, distortion and minimum TE, which are not included in the model, may favour the adoption of a lower b-value than the theoretical optimum.

## O4 Preliminary evaluation of the impact of social media advertising on citations of medical imaging publications

### Gelardi, A F Barrero, G Converso, S Di Giorgio, M C Grondelli, B Maizza, S M Tarchi, M Sollini, N Gozzi, A Chiti

#### ^1^Department of Biomedical Sciences, Humanitas University, Pieve Emanuele, Milan, Italy; ^2^ IRCCS Humanitas Research Hospital, Rozzano – Milan, Italy, 1. Department of Biomedical Sciences, Humanitas University, Pieve Emanuele, Milan, Italy

##### **Correspondence:** Gelardi (fabrizia.gelardi@humanitas.it)

Background: The use of social media is increasing exponentially. Following this trend, scientific journals and authors attempted to increase the visibility of their papers exploiting social media. We aim to evaluate the impact of social media advertising on citations of medical imaging publications.

Material and Methods: We selected on Embase papers published in 2018 on six journals (“European Radiology”, “Radiology”, “Journal of Nuclear Medicine”, “European Journal of Nuclear Medicine and Molecular Imaging”, “Radiotherapy and Oncology” and “International Journal of Radiation Oncology, Biology and Physics”). Public interest for each paper was measured by Scopus and Altmetric, including number of citations, Altmetric score and its details. Two subsets were defined based on citations. Correlation between variables was assessed by Spearman and Kendall’s correlation coefficients. Random Forest (RF) and Boosted Regression Tree (BRT) models were employed to investigate the relationship between citations and all correlated covariates. Analyses were performed on R and Python.

Results: A significant correlation between citations and several predictors was found, including Mendeley (p-value=0.0003). Low-citations subset significantly correlated with several predictors, including readings on Mendeley, number of Twitter and Facebook accounts. BRT achieved a root-mean-square deviation of 17.40 with the residuals increasing exponentially with citations. Moreover, Mendeley, number of Twitter accounts and the sum of all ‘cited by’ entries were highly influential on citations.

Conclusions: Publications’ advertising may partially influence the number of citations. A new metric based on proven significant predictors can be established to anticipate the number of citations. However, citations exceeding a certain threshold may be a challenge to predict.

## O5 Efficacy, safety and advantages of Ultrasound guided biopsy of pleural and peripheral lung lesion

### Binoy Choudhury (francy-cast@hotmail.it)

#### Dr B Borooah Cancer Institute, Guwahati, India

Aim : Ultrasound (US) guided needle biopsy is a very useful, easily available, less expensive, safer diagnostic technique with real-time monitoring The purpose of this study was to determine the efficacy, safety and advantages of US guided biopsy of pleural and peripheral lung lesions abutting pleura and to compare with CT guidance.

Materials and Methods : Among 1932 image guided thoracic biopsies obtained at our hospital between January 2001 and December 2019, 355 were US guided biopsies for pleural and peripheral lung lesions (272-male, 83-female). Of these, 335 were performed using US only; the remaining 20 had initial CT localization. There were 25 pleural lesions and 330 pulmonary lesions with pleural contact. After reviewing the patient, CT scan and coagulation profile, biopsy was performed using free hand US technique under real-time visualization. Lesion size, lesion pleural contact, biopsy type, number of passes, procedure time, sample adequacy and complications were recorded.

Results : Among 1932 biopsies, 355 were US guided (18.37%) and 1577 (81.63%) were CT guided procedures. Procedure time was significantly less in US guidance than under CT guidance. Post procedure pneumothorax was significantly less in US guided group as observed in one of 355 US guided procedures (0.28%) and 41 of 1577 CT guided procedures (2.6%). Intraparenchymal hemorrhage occurred in one of 355 US guided biopsies (0.28%) and 14 of 1577 under CT guidance (0.89%).

Conclusions : US guidance allows significantly less procedure time and post-procedural complications without the use of ionizing radiation

## O6 RECIST 1.1 Target Lesion Categorical Response in Metastatic Renal Cell Carcinoma: Conventional versus Volumetric Assessment

### A. Gong, K. Ruchalski, H. J. Kim, M. Douek, A. Gutierrez, M. Patel, V. Sai, H. Coy, B. Villegas, S. Raman, J. Goldin

#### David Geffen School of Medicine, University of California, Los Angeles, CA, USA, UCLA, Department of Radiological Sciences, Los Angeles, CA, USA; UCLA Centre for Computer Vision and Imaging Biomarkers, Los Angeles, CA, USA

##### **Correspondence:** A. Gong (ajgong@mednet.ucla.edu)

Aim: Response Evaluation Criteria in Solid Tumours version 1.1 (RECIST 1.1) relies on single tumour diameter measurements, approximating spherical tumours and isotropic change. This study characterises the effect of RECIST approximations by comparing categorical response assessment determined by a spherical model of tumour volume versus actual tumour volume.

Materials & Methods: This retrospective study of metastatic renal cell carcinoma includes patients with ≥1 target lesion (TL) at baseline and at least one follow-up CT chest, abdomen, and pelvis. TL volume was assessed by 1) Vmodel, spherical approximation extrapolated from RECIST diameter and 2) Vactual, manually contoured volume. Volumetric categorical responses (thresholds extrapolated from RECIST) were determined with the sum of TL volumes per patient. Percent-error (Vmodel-SUM versus Vactual-SUM) reflects RECIST’s error in approximation of tumour burden. Paired proportion test and McNemar test compared categorical response

Results: TLs were assessed at baseline (N=638), week 9 (N=593), and week 17 (N=508). Overestimation of tumour burden by Vmodel does not correlate with actual change in tumour burden, Vactual (p=0.36 week 9 and p=0.72 week 17). However, when categorical thresholds are applied there is a significant difference between volumetrically derived and actual volume categorical classification (p<0.0001). Significantly more patients were categorised as having progressive disease by Vmodel (p=0.001) which would suggest a detected difference in therapeutic treatment failure.

Conclusions: Actual contoured tumour volumes result in a more conservative categorical response classification compared to the volumetric model of RECIST, suggesting that RECIST spherically derived volumes leads to overestimation of tumour burden, resulting in significant response discordance and over classification of disease progression.

## O7 Preoperative [11C]methionine PET for the assessment of molecular subtype and prognosis in grade II/III gliomas

### G Ninatti, M Sollini, B Bono, N Gozzi, D Fedorov, L Antunovic, F Gelardi, F Pessina, A Chiti

#### Humanitas University, IRCCS Humanitas Research Hospital, Milan Italy

##### **Correspondence:** G Ninatti (gaia.ninatti@gmail.com)

Aim: PET with radiolabelled amino acids is used in the preoperative evaluation of patients with glial neoplasms. This study aimed to assess the role of [11C]methionine (MET) PET in differentiating molecular subtypes and predicting prognosis in newly-diagnosed grade II/III gliomas surgically treated.

Materials & Methods: Patients with a new diagnosis of grade II/III glioma who underwent surgery at our Institution and were imaged preoperatively using [11C]MET-PET/CT were retrospectively included in the study. [11C]MET-PET images were qualitatively and semi-quantitatively analyzed using tumour-to-background ratio (TBR). Progression-free survival (PFS) rates were estimated using the Kaplan-Meier method and Cox proportional-hazards regression was used to test the association of clinico-pathological and imaging data to PFS.

Results: 201 patients with grade II/III glioma met the inclusion criteria. Overall, 150 lesions (75%) were positive, while forty (20%) and eleven (5%) were isometabolic and hypometabolic at [11C]MET PET, respectively. [11C]MET uptake was more common in oligodendrogliomas and grade III IDH-wildtype astrocytomas compared to IDH-mutant and grade II IDH-wildtype astrocytomas (87% and 93% vs. 50% and 63% of cases, respectively). Among [11C]MET-positive gliomas, grade III IDH-wildtype astrocytomas and grade III oligodendrogliomas had the highest median TBRmax (4.65 and 3.22, respectively). In IDH-mutant astrocytomas, higher TBRmax values at [11C]MET PET were independent predictors of shorter PFS.

Conclusions: This work highlights the role of preoperative [11C]MET-PET in estimating the molecular subtype of grade II/III gliomas and predicting their biological behaviour and prognosis. Our findings support the implementation of [11C]MET-PET in routine clinical practice for the better management of these neoplasms.

## O8 FDG PET/CT scan in characterisation of solitary pulmonary nodules

### Abtin Doroudinia, Mehrdad Bakhshayesh Karam, Azam Alavi

#### Chronic Respiratory Diseases Research Center, National Research Institute of Tuberculosis and Lung Diseases (NRITLD), Shahid Beheshti University of Medical Sciences, Tehran-Iran

##### **Correspondence:** Abtin Doroudinia (abtin1354@gmail.com)

Aim: Diagnosis of malignancy versus benignity of solitary pulmonary nodules (SPNs) is critical, considering significant outcome differences. Histopathologic surgical evaluation is the gold standard method and due to its invasive nature, other diagnostic modalities such as PET/CT scan has also been considered.

Material and methods: In this cross-sectional study, we evaluated SPNs in 56 patients with 18F-Fluorodeoxyglucose (FDG) PET/CT scan. Following PET/CT evaluation, pulmonary nodule sampling and definitive diagnosis based on histological evaluation was established. Finally, the diagnostic value of FDG PET/CT scan compared with histopathological evaluation was determined.

Results: Based on histopathological findings, 32 cases had malignant SPN (57.1%) and 24 cases were benign (42.9%). The mean value of SUVmax marker was 4.35±4.41. Considering PET imaging criteria (1 to 5 scale grading based FDG uptake on PET images compared to the liver, considering grade 3 or higher as malignant), 34 cases (60.7%) were classified as malignant nodules and 22 cases (39.3%) were benign. Compared to the histopathological findings as the standard for diagnosis, FDG PET technique had 100% sensitivity, 91.6% specificity, 94.1% positive predictive value (PPV), 100% negative predictive value (NPV) and 96.4% diagnostic accuracy. Considering the value of SUVmax, measurement of this index was found to be highly valuable in distinction between malignant and benign nodules. Higher values of SUVmax (more than 1.6) had 96.9% sensitivity and 75.0% specificity to predict malignant nodules.

Conclusion: FDG PET/CT scan with high sensitivity and specificity can make distinction between malignant and benign solitary pulmonary nodules and higher SUVmax values are a significant surrogate of malignancy in SPNs.

## O9 Transfer learning applied to medical imaging: a proof-of-concept study on chest radiographs

### F Gelardi^1^, N Gozzi^2^, E Giacomello^3^, M Sollini^1, 2^, M Kirienko^4^, C Jones^5^, P Lanzi^3^, D Loiacono^3^, A Chiti^1, 2^

#### ^1^Department of Biomedical Sciences, Humanitas University, Pieve Emanuele, Milan, Italy; ^2^IRCCS Humanitas Research Hospital, Rozzano – Milan, Italy; ^3^Dipartimento di Elettronica, Informazione e Bioningegneria, Milan, Italy, 1. Department of Biomedical Sciences, Humanitas University, Pieve Emanuele, Milan, Italy; ^4^Fondazione IRCCS Istituto Nazionale Tumori, Milan, Italy; ^5^Dipartimento di Biotecnologie Mediche e Medicina Traslazionale, Università degli Studi di Milano, Milan, Italy

##### **Correspondence:** F Gelardi (fabrizia.gelardi@humanitas.it)

Introduction: Transfer learning is an effective approach to accelerate training and increase CNN performance on small datasets exploiting pre-trained models on independent larger datasets. However, in medical imaging pre-trained machine learning lacks models. We aim to develop CNN models to extract features, pre-trained on large public datasets of labeled chest radiographs (CXRs), and to classify image embedding using tree-based models on an independent local dataset.

Methods: Seven CNNs were pre-trained on CheXpert using a public dataset of 223316 CXRs with 14 major-findings labels. Pre-trained networks were applied to extract features on an independent dataset of 941 CXRs performed at IRCCS Humanitas Research Hospital. Each image was labeled as normal/abnormal and abnormalities were further classified as cardiac, lung, pneumothorax, pleura, bone, device. Each CNN output (image embeddings) was extracted before the classification layer and a Random Forest (RF) model was trained to perform multi-label classification. Results were pooled using simple and entropy-weighted averaging. Model performance was assessed through the area under the curve (AUC).

Results: The best RF model was obtained with simple averaging of the predictions. AUC values achieved for each label were: normal 0.86, cardiac 0.85, lung 0.72, pneumothorax 0.92, pleura 0.94, bone 0.85, device 0.86 (average of 0.856). The classification time was less than 1s.

Conclusions: Image embeddings extracted from CNNs pre-trained on public datasets can be exploited by a tree-based classifier on an independent dataset with good performance. Transfer learning approach can be applied to medical imaging, overcoming the requirement of large private datasets, high computational resources and long training sessions.

## P1 Conventional MRI-based radiomics analysis for discriminating salivary gland tumours

### Rongli Zhang, Qi Yong H. Ai, Lun M. Wong, Sahrish Qamar, Ann D. King

#### Department of Imaging and Interventional radiology, Faculty of Medicine, The Chinese University of Hong Kong, Shatin, Hong Kong S.A.R.

##### **Correspondence:** Rongli Zhang (1155154037@link.cuhk.edu.hk)

Aim: This study aimed to investigate which conventional MRI sequence performed best for radiomic analysis in discriminating malignant tumours (MT), pleomorphic adenomas (PA) and Warthin tumours (WT).

Methods: Axial T1-weighted images (T1WI), fat-suppressed T2-weighted images (FS-T2WI), and contrast-enhanced (CE)-T1WI of patients with salivary gland tumours (n = 94; 34 MT, 44PA, 16 WT) were evaluated. Each sequence was split into training (70%) and testing sets (30%) and data augmentation was performed using the AUSWO technique in training and testing sets. 1015 radiomics features were extracted from each MRI sequence and feature selection was performed using the LASSO algorithm with 10-fold cross-validation. Multivariable logistic regression was used to construct the models using selected features from each MRI sequence to discriminate MT from PA and WT, respectively. Each model performance was evaluated with 500 times bootstrap in the testing set.

Results: For discriminating MT from PA, the model constructed using nineteen selected features from FS-T2WI showed the best performance in the testing cohort (area under the curve [AUC] 0.953, accuracy 96.2%, sensitivity 92.4% and specificity 100.0%) when compared to the other models (P values<0.001). For discriminating MT from WT, the model constructed using two selected features from T1WI showed the best performance in the testing cohort (AUC 0.705, accuracy 75.6%, sensitivity 90.4% and specificity 60.8%), when compared to the other models (P values<0.001).

Conclusion: Radiomic analysis of MRI for discrimination of salivary gland tumours did not require a contrast enhanced sequence in this study.

## P2 Post-transplant Lymphoproliferative Disorder (PTLD) in Children: About two cases

### G Ficalora, A Sabatella, A Monjes, S De Luca

#### Hospital Alemán, Buenos Aires, Argentina

##### **Correspondence:** A Sabatella (agustinasabatella@gmail.com)

Learning Objectives:

- Analyse risk factors and epidemiology of PTLD, along with the virology and tumoural biology in the evolution of it
- Describe imaging findings and the role of the different diagnostic imaging methods in this pathology
- Mark out the imaging mimics that overlap with PTLD and the possible differential diagnosis

Content Organisation:

Post-transplant lymphoproliferative disorder (PTLD) is the most common malignant neoplasia in post-transplanted children. The aetiopathology and risk factors associated with this disease are not clearly known yet. However, Epstein-Barr virus has been identified in many patients with PTLD, contributing to its development. The different imaging methods at our disposal (US, CT, MRI, PET-CT), are key elements in diagnosis, analysis of therapeutic response and in the follow-up of this disorder. This pathology has a high mortality rate when the diagnosis is not appropriate or swift enough. This is why the radiologist must be aware of its existence and be able to interpret the different imaging findings, especially in the first year post-transplant, facilitating decision making about patient treatment. We will present two cases of transplanted infants who developed PTLD, approximately one year after their surgeries.

Conclusion:

The different imaging methods are essential in the diagnosis of PTLD, aiding as well, in the staging, the assessment of therapeutic response and long term surveillance. Familiarity with its imaging findings optimises rapid diagnosis, improving the chances of good disease management and a good outcome.

## P3 Pitfalls in RECIST 1.1 in Clinical Trials

### Renn A, Hopkinson G, Kafaei L, Tunariu N, Sohaib A

#### The Royal Marsden Hospital, Sutton, UK

##### **Correspondence:** Renn A (alexandra.renn@rmh.nhs.uk)

Learning Objectives

1. Application of RECIST 1.1 in a clinical trial setting
2. Understand the limitations of RECIST 1.1 when evaluating disease response
3. Highlight areas and illustrate potential difficulties in assessing response

Content Organisation

1. Background to RECIST 1.1: Briefly outline the current RECIST 1.1 guidelines and how to assess response.

2. Illustrative examples, with succinct explanation of the following pitfalls experienced in our clinical practice:

i) **Mixed solid/ cystic lesions**: the enlarging ‘cystic’ component in mixed solid/cystic lesions and ‘necrotic’ tumour change

ii) **The ‘obscured’ lesion:** target lesions which become non-visible, e.g. lung tumours with overlying consolidative change

iii) **Isolated lesions treated with radiotherapy**

iv) **New sclerotic bone lesions**: treated disease versus potential new lesion

v) **Serosal bowel disease**: difficulty in selecting such lesions as target, and subsequently assessing response

vi) **Lymph nodes**: Clinical progression versus RECIST 1.1 progression, e.g. a new lymph node measuring 12mm in its short axis.

vii) **Calcified lesions**: Can there ever be complete response if the lesion(s) subsequently calcifies?

viii) **The new synchronous primary**: Does this equate to progressive disease?

3. Ensuring reproducibility- Inconsistencies of lesion definition/measurements in follow-up. Is there a role for standardised reporting software?

Conclusion

RECIST plays a vital role in tumour assessment in the clinical trial setting, and one has to be aware of its use to apply it correctly. Limitations of RECIST 1.1 impair subsequent disease assessment. The radiologists should be aware that even minor mistakes of RECIST 1.1 application can dramatically influence treatment decision and patient outcome.

## P4 Preventing and minimizing the risk of Pneumothorax in Image Guided Percutaneous Thoracic Biopsy

### Binoy Choudhury (choudhury60@gmail.com)

#### Dr B Borooah Cancer Institute, Guwahati, India

Learning objectives :

1.To review various factors associated with risk of post thoracic biopsy pneumothorax

2.Illustrate the uses of various methods and techniques to prevent and minimise the risk of pneumothorax in image guided percutaneous thoracic biopsy.

Content organisation:

Introduction:

Pneumothorax is the most common complication of any thoracic biopsy. Pneumothorax requiring chest tube and hospital admission is a painful and distressing experience usually judged by the patient and referring clinician.

Factors associated with post-biopsy pneumothorax:

Chronic obstructive pulmonary disease, bulla & emphysema in the needle path, small size lesions, deep seated lesions, longer procedure time, multiple needle passes through pleura and lung, less experienced operator, use of large bore needle, etc.

Various methods and techniques to minimise pneumothorax:

Co-axial needle technique (single pleural puncture), use of small bore needle, restriction of coughing, talking, deep breathing & other activities during the procedure, avoiding pleural puncture during local anaesthesia, major adjustment of needle trajectory within the chest wall, use of sterile towel to keep the needle in intended position, use of ultrasound guidance for pleural or peripheral lung lesions abutting pleura, minimizing pathway of normal lung, pathway through non-aerated lung, extrapleural parasternal and paravertebral approach, widening of extrapleural space by saline injection, transsternal approach, avoidance of bullae, bronchi and fissures, biopsy through consolidated area or attached segment of lesion with pleura, autologous blood patch technique, immediate dependent positioning of the puncture site.

Conclusions**:**

Risk of pneumothorax can be significantly minimised and prevented by various methods and techniques.

## P5 An Unusual CT Appearance of Lung Metastasis mimicking COVID-19 Infection

### Ahmad NA^1^, Brama Kumar J^1^, Abdul Hamid Z^1^, Marjan N^2^

#### ^1^ Institut Kanser Negara, Putrajaya, Malaysia; ^2^ Hospital Putrajaya, Malaysia

##### **Correspondence:** Ahmad NA (azlinahmad@gmail.com)

Current literature describing chest CT abnormalities of COVID-19 pneumonia frequently report high incidence findings as ground glass opacities with bilateral and lower lobe predominance. These findings were seen in more than 70% of patients with positive RT-PCR proven COVID cases. Patients exhibiting these findings in chest CT should be suspected of having COVID-19 infection. However, in some rare cases, imaging of atypical lung metastasis may also mimic infection such as COVID-19. Our patient is a lady who was diagnosed with squamous cell carcinoma of the cervix and is not known to have lung metastasis on staging CT. Soon after diagnosis, she presented with a two-week history of fever, cough and shortness of breath. CTPA revealed bilateral lung ground glass opacities with lower lobe predominance as well as segmental pulmonary vasculature irregularity highly suspicious of COVID-19 pneumonia. The patient expired soon after the CTPA. RT-PCR taken immediately following her death was negative for COVID-19. Post-mortem histopathology revealed numerous foci of malignant tumour clusters in the vessels, bronchioles and alveolar spaces in keeping with diffuse lung metastasis. This case illustrates an atypical CT appearance of lung metastasis in a patient with squamous cell carcinoma of the cervix, similarities of COVID-19 pneumonia on chest CT to other entities as well as the challenge faced by radiologists when interpreting chest CT in the oncologic subpopulation particularly when clinical presentation is also unusual.

Written informed consent was obtained from the patient’s next of kin for writing of this case report.

## P6 Breast Implant-Associated Anaplastic Large Cell Lymphoma: Current diagnostic and management guidelines in the UK and USA

### M Adejolu, T Gagliardi, B Sharma

#### The Royal Marsden Hospital, London UK

##### **Correspondence:** M Adejolu (margaret.adejolu@rmh.nhs.uk)

Learning Objectives

1. To describe the epidemiology, pathogenesis and clinical presentation of BIA-ALCL subtypes
2. To review multimodality imaging findings of BIA-ALCL highlighting common clinical diagnostic dilemmas
3. To review and compare recently published UK and USA guidelines on the diagnosis and management of BIA-ALCL

Content Organisation:

Breast implant–associated anaplastic large cell lymphoma (BIA-ALCL) is a malignancy recently recognised by the World Health Organization Classification of lymphoid tumours. The condition, and imaging findings, are highly nuanced compared with other lymphomas and solid cancers. Patients can be prone to delayed diagnosis; data-interpretation, both in terms of pathology and multiparametric imaging is challenging. We will review and compare recent published guidelines from the UK and USA in the diagnosis and evaluation of BIA-ALCL and discuss the pivotal role of multimodality imaging.

1. Epidemiology, clinical presentation, clinical course, and prognosis of BIA-ALCL
2. Role of Radiology in the diagnosis and management of BIA-ALCL
  - Ultrasound
  - Mammography
  - Magnetic Resonance Imaging
  - FDG PET/CT
  - Staging CT
3. Tissue sampling and histopathological assessment
4. Diagnostic Pathways and guidelines
  - The UK diagnostic algorithm
  - The USA diagnostic algorithm
5. Management guidelines in the UK and USA
6. Disease surveillance: Review of UK and USA recommendations

Conclusion:

Recent consensus guidelines on the diagnosis and management of BIA-ALCL produced in the UK and USA are helping to standardise diagnosis and management. Radiologists working in breast imaging, lymphoma and various radiological disciplines will find the nuanced features presented useful to avoid diagnostic delays, enable best use of imaging resources and achieve best outcomes for patients.

## P7 Extranodal marginal zone lymphoma (ENMZL) location, CT and PET/CT role: our experience

### C Carrera, M Escolar, C Frias, C Congost, M Saborido, G Rodriguez, Y Troncoso, S De Luca

#### Hospital Alemán, Buenos Aires, Argentina

##### **Correspondence:** M Escolar (mescolar@hospitalaleman.com)

Learning objectives:

Show different locations of presentation of lymphomas (ENMZL). Describe the most typical imaging findings on CT and in some cases their correlation with PET/CT studies. Identify the role of CT and PET/CT in their diagnosis, evaluation and staging.

Content organisation:

Extranodal marginal zone B lymphoma (ENMZL), formerly known as mucosa-associated lymphoid tissue lymphoma (MALT), is a rare form of malignant non-Hodgkin's lymphoma, which affects the development of B-cells at the expense of lymphoid tissue related with mucous membranes. Can be developed in the intestinal tract (especially stomach), lungs, and glands (lacrimal, thyroid, and mammary). Although it can involve, more rarely, the lymph nodes. The definitive diagnosis is based on the histology of the lesion and endoscopic examination is necessary in the case of gastrointestinal or pulmonary lymphomas. CT and PET / CT can determine the stage of the disease. We present 3 cases of ENMZL located in the small bowel, lacrimal gland and less frequent lungs. Confirmation was made after surgery and/or biopsy and subsequent pathological study. The presentation was as a single or multiple nodule/mass, with a definitive diagnosis after removal.

Conclusions:

The manifestations of ENMZL can mimic a variety of diseases in tomography, however, certain radiological characteristics should persuade the suspicion of a lymphoma as a differential diagnosis, without forgetting the importance of histology in the definitive diagnosis. CT is the most useful modality because it provides better general assessment of the stage of the disease. A close diagnosis can lead to early treatment and prevent unnecessary surgeries.

## P8 Clear cell Likelihood Score (ccLS) V2.0 in cT1a renal masses on MRI: radiologic-pathologic correlation

### Y Troncoso, L Alvarez, M Nazar, A Ramirez, P Insaurrald, S De Luca

#### Hospital Alemán, Buenos Aires, Argentina

##### **Correspondence:** L Alvarez (leandroalvarez85@hotmail.com)

Learning objectives:

- Apply ccLS in small renal masses

- Evaluate the diagnostic performance of multiparametric MRI in the diagnosis of clear cell histology in cT1a masses

- Describe the radiologic findings in solid renal masses

- Illustrate an imaging and pathology correlation of the most common solid renal masses

Content organisation:

The clear cell renal carcinoma probability score analyzes renal masses smaller than 4 cm - T1a. Macroscopic fat tumours (corresponding to AML) and cystic tumours with less than 25% solid component (Bosniak would apply) are not included.

Lesions are classified in clear cell likelihood score: 1) definitely not, 2) probably not, 3) equivocal, 4) probably, and 5)definitely, being ccLS, 1 less likely and ccLS, 5 more likely to correspond to clear cell renal carcinoma.

The diagnostic algorithm is based on major criteria like T2-weighted (hyperintense – isointense - hypointense), contrast enhancement characteristics (intense, moderate, mild) and if microscopic fat present. In some cases it is necessary to use ancillary findings to select the most likely diagnosis (arterial-to-delayed enhancement ratio, if segmental enhancement inversion is present, if it is homogeneous or heterogeneous and marked restriction on DWI).

We selected representative cases in order to apply the score to determine the most probable diagnosis and compare it with the histopathological results.

Conclusion:

The ccLS offers the possibility of standardizing the interpretation of small renal masses. It could also guide decision-making in these patients and reduce the number of diagnostic renal mass biopsies.

## P9 Hereditary renal tumours: an overview, imaging features and implications for management

### K Paramesh^1^, A Gupta^2^, H Verma^1^

#### ^1^ Guys and St Thomas NHS Trust, London, UK; ^2^ Royal Sussex County Hospital, University Hospitals Sussex NHS Foundation Trust, Brighton, UK

##### **Correspondence:** K Paramesh (akshatha_p@yahoo.com)

Aim**:**

Hereditary renal tumours: an overview, imaging features and implications for management

Learning Objectives:

To review the spectrum of imaging findings of hereditary renal tumours and associated implications for management.

Content Organisation:

Hereditary renal tumours are often suggested by family history, age of onset and the presence of other lesions typical for respective syndromes. 5-8% of RCC are hereditary and to date, 10 hereditary RCC syndromes are known with specific germline mutations, RCC histology and co-morbidities.

We will present the spectrum of imaging features of hereditary renal tumours in the following conditions:

Von Hippel Lindau syndrome

Tuberous Sclerosis

Lynch syndrome (hereditary non-polyposis colorectal cancer syndrome)

Burt-Hogg Dube syndrome

Hereditary pRCC

Hereditary leiomyomatosis and RCC (HLRCC)

Succinate dehydrogenase (SDH) mutation

Phosphatase and tensin homolog (PTEN) hamartoma syndrome

Renal medullary carcinoma (associated with hereditary haemoglobinopathies)

Diagnosis of a hereditary basis for renal tumours has multiple implications for management. This includes appropriately timed nephron-sparing approaches, active surveillance taking into account growth kinetics, size and location of tumours and screening for renal and extra-renal lesions.

Conclusion:

Recognition of hereditary renal tumours is important as patients and their families are at increased risk of renal and extra-renal malignancies at a younger age. Diagnosis of a hereditary basis has significant implications for planning management to avoid repeated surgical interventions, morbidity and mortality.

## P10 Gastrointestinal and extra-gastrointestinal stromal tumours : What the radiologist needs to know

### K Paramesh, N Griffin

#### Guys and St Thomas' NHS Trust, London, UK

##### **Correspondence:** K Paramesh (akshatha_p@yahoo.com)

Learning objectives:

To review common and unusual presentations of GISTs and extra-GISTs. To understand the pivotal role of radiology in diagnosis, staging and management.

Content organisation:

GISTs are the most common mesenchymal tumours of the gastrointestinal tract, most commonly located in the stomach and small intestine. They can present less frequently in the rectum and oesophagus and other intra-abdominal soft tissues. The latter are known as extra-gastrointestinal stromal tumours (extra-GISTs) and occur in the omentum, mesentery or retroperitoneum.

GISTs and extra-GISTs may be sporadic or associated with inherited syndromes, notably in neurofibromatosis-1 and Carney triad. Patients usually have gastrointestinal bleeding, anaemia, abdominal pain, but haemorrhage, tumour rupture and bowel perforation may be seen in late presentations. When diagnosed and treated appropriately, these tumours have an excellent prognosis, and as such radiologist familiarity is important.

We will review:

Common and unusual locations of GISTs and extra-GISTs in different modalities

Differential diagnoses to consider depending on the location

Importance of imaging based diagnosis

Pre-operative staging

Patterns of metastatic spread

Complications

Post-operative surveillance

Conclusion:

GISTs and extra-GISTs are the most common mesenchymal tumours of the abdomen and have an excellent prognosis when detected and treated in a timely manner. Therefore, a good understanding of all imaging aspects of the disease is pivotal to ensure a good patient outcome.

## P11 BRAF V600E-mutated Colorectal Cancer: A Pictorial Review

### K Paramesh, N Maisey, J deNaurois, P Ross, F Chinaka

#### Guys and St Thomas' NHS Trust, London UK

##### **Correspondence:** K Paramesh (akshatha_p@yahoo.com)

Aim**:**

BRAF V600E-mutated Colorectal Cancer: A Pictorial Review

Learning objectives:

To review the imaging findings in BRAF V600E-mutated colorectal cancers and understand the implications for management

Content organisation:

BRAF mutations occur in a minority of colorectal cancers cases (5-15%) compared to KRAS mutations in codon 12 and 13 (40%) and extended KRAS and NRAS mutations (52%). Among other malignancies, BRAF-V600E mutation occurs in melanoma (66%), papillary thyroid cancer (53%) and serous ovarian cancer (30%). However, when present, BRAF V600E-mutated colorectal cancers are associated with worse outcomes. These tend to present on the right side and in women older than 60 years old. They have a unique metastatic pattern with more frequent nodal and peritoneal disease and ascites at the time of presentation. Due to their aggressive nature, only 60% of patients can receive second-line chemotherapy, so early recognition and initiation of a tailored first-line approach with kinase inhibitors such as encorafenib and monoclonal antibodies such as cetuximab may be an effective strategy.

We will review:

Clinical features

Histopathological correlation

Typical imaging findings of the initial presentation

Radiological challenges of measuring therapeutic outcomes

Implications on management

Conclusion:

BRAF V600E-mutated colorectal cancers are a relatively rare finding but often present with characteristic clinical features, histopathology and imaging findings. Given its association with negative outcomes, early recognition and use of targeted therapy has important implications for patient outcome.

## P12 Demystifying mucinous ovarian tumours: MRI characteristics of primary and metastatic mucinous ovarian neoplasms

### V Mellou, A Azam, E Ainsworth, A Mayers, J Cleary, S Hasso, A Jacques, O Westerland, S Natas

#### Guy’s and St Thomas’ NHS Foundation Trust, London, UK

##### **Correspondence:** V Mellou (vickmell7@gmail.com)

Learning Objectives:

To present the imaging spectrum of the different subtypes of primary and secondary mucinous ovarian neoplasms to help guide optimal patient treatment.

Content Organisation:

Primary mucinous ovarian tumours represent a subset of epithelial neoplasms. However, these tumours may also be secondary to metastases from other adenocarcinoma primaries, usually arising from the gastrointestinal tract.

Primary mucinous ovarian tumours are classified into:

- Benign ovarian mucinous cystadenoma
- Ovarian borderline mucinous tumour
- Malignant ovarian mucinous cystadenocarcinoma

Metastases most commonly result from: appendix, colon, stomach, gallbladder or pancreatic primaries.

We will highlight the MRI features of primary mucinous ovarian tumours, demonstrating benign, borderline and malignant neoplasms, and will provide examples of metastases, and their primary disease. Accurate assessment at diagnosis is vital to help guide optimal management, whether by limited surgery, a full ovarian cancer staging operation, or systemic treatment, in the case of metastases. We will also highlight the importance of surveillance after initial treatment, with a case of malignant transformation of peritoneal implants after primary mucinous borderline ovarian tumour.

Conclusion:

MRI allows accurate characterisation of ovarian mucinous lesions to allow differentiation of benign, borderline and malignant primary tumours, and imaging features may suggest an alternate primary tumour, which may be identified on review of the remainder of the abdomen and pelvis. Cross sectional imaging is also important for post-operative surveillance.

## P13 Renal Involvement in Lymphoma; extranodal high risk lymphoma sites for secondary CNS lymphoma and the SIHMIR tool

### E. Musanhu, B. Sharma

#### The Royal Marsden Hospital, Sutton, UK

##### **Correspondence:** E. Musanhu (ed.musanhu@oxon.org)

Learning objectives:

To review the imaging patterns of renal involvement in lymphoma.

Content organisation: Recognition of renal lymphoma involvement in DLBCL patients is critical, the kidneys being a paired organ high risk extranodal risk site for secondary CNS lymphoma. The number of involved extranodal sites is critical for prognostic score modelling defining whether CNS prophylaxis therapy is required (testes, adrenals, kidneys and breasts being high-risk sites). Nuanced features of renal lymphoma on CT and PET are presented, together with features of other extranodal risk sites, prognostic risk models for secondary CNS lymphoma (SCNSL) and a ‘specialist integrated haematological malignancy imaging reporting’ (SIHMIR) methodology for clinical and research practice in lymphoma radiology. We present examples of patterns of renal involvement encountered on CT and PET:

- Focal cortical lymphomatous lesions, nuances on CT, PET imaging, and of response assessment. High grade lymphomas – DLBCL.

- Infiltrative disease with no discrete mass

- Solitary masses (often difficult to differentiate from the hypovascular papillary and chromophobe RCC subtypes)

- Contiguous spread from retroperitoneal disease

- Pararenal fascia lymphoma infiltration- low grade NHLs, e.g. extranodal marginal zone lymphoma, nuances on CT and PET

- Pelvicalyceal, ureteric and bladder lymphoma - typically follicular lymphoma

- Extranodal high risk sites for secondary CNS lymphoma (SCNSL; testes, adrenals, kidneys, breasts)

- Prognostic risk models for SCNSL.

- Specialist integrated haematological malignancy imaging reporting’ (SIHMIR) methodology (template driven tool).

Conclusions: Recognition of extranodal involvement is important for proper prognostication and clinical management.

## P14 Chronic Lymphocytic Leukaemia and Richter Transformation, an overview of multiparametric imaging principles, diagnosis and pitfalls

### E. Musanhu, B. Sharma

#### The Royal Marsden Hospital, Sutton, UK

##### **Correspondence:** E. Musanhu (ed.musanhu@oxon.org)

Learning objectives:

To review and outline the role of imaging in the management of Chronic Lymphocytic Leukaemia (CLL) and Richter Transformation (RT).

Content organisation:

CLL is the commonest leukaemia amongst adults in the Western world. The incidence is increasing in tandem with the aging population with a concomitant RT increase expected as patients survive longer owing to the novel therapies

Whereas CLL diagnosis and surveillance is predominately clinical with the aid of haematology, imaging plays a pivotal role in early recognition of possible RT, a critical complication of CLL with poor patient outcomes requiring urgent change of therapy.

We will present the spectrum of imaging findings covering the following:

- CT and PET/CT appearances of indolent & uncomplicated CLL
- Accurate splenic evaluation on CT in CLL patients
- CT and PET/CT features suggestive of accelerated phase CLL and RT
- Diagnosis of RT – US-guided biopsy and use of multiparametric imaging for selection of biopsy sites
- Histological appearances of the two main RT variants
- Potential pitfalls in the diagnosis of RT including its mimics and the effect of novel immune therapy agents on imaging manifestations of RT.

Conclusions:

It is imperative that radiologists and clinicians are aware of the nuances of multiparametric imaging in CLL, the natural history of CLL and RT, and are able to diagnose RT in a timely manner to improve patient outcomes. An algorithm for the use of PET/CT as a decision-making tool in CLL, accelerated phase CLL and RT is presented, with regard to a) whether biopsy is indicated, and b) selection of a representative biopsy site.

## P15 Accuracy of F18-FDG-PET/CT in pretherapeutic lymph node staging in NSCLC: A single-center retrospective analysis

### V. von Thun, J. Müller-Hübenthal, J. Sommerlath-Sons, M. Hoffmann

#### Praxis im KölnTriangel Germany, Praxis im KölnTriangle Germany, Praxisnetz Radiologie Bonn Germany, Department of Occupational Health & Safety Bonn Germany

##### **Correspondence:** V. von Thun (vickyvonthun@hotmail.com)

Aim: To evaluate the incremental performance of FDG-PET/CT in preoperative lymph node staging of NSCLC based on an outpatient single centre database.

Method: Out of a total of approximately 5870 examinations from 2007-2021, integrated FDG-PET/CT examinations were performed according to EANM procedure guidelines and EARL performance standard for Initial staging in n=375 patients with newly diagnosed non-small cell lung cancer. Standardised reports were generated according to TNM-7 and TNM-8 respectively. Lymph node staging was pathologically confirmed in all patients on surgical specimen, TBNA, EBUS and/or mediastinoscopy. Pre-PET/CT staging was based on CT-scans in all patients. Lymph nodes in FDG-PET/CT were considered positive if corresponding focal FDG-uptake was equal or higher than mediastinal-blood-pool level.

Results: In the current analysis integrated PET/CT correctly N-staged 260 out of 375 patients (69,3%). Pre-PET/CT N-Staging based on CT scan and clinical appearance remained unaltered in 42% of the cases. Upgrade of the N-stage resulted in 40% of the cases, and downgrade in 7%, histologically confirmed in 23% and 3.2% respectively. Sensitivity and specificity of PET/CT were 85% and 75%. 7% of cases were falsely staged N0. Possible reasons were direct tumour infiltration, low uptake, neoadjuvant chemotherapy but predominantly truly PET negative cases.

Conclusion: These data obtained from a private outpatient setting are in agreement with the published literature as well as current guidelines and confirm an incremental value of integrated PET/CT to CT alone in pretherapeutic N-staging of NSCLC patients. However the primary scope of PET/CT is M-staging. Reasons for false negative PET/CT reports are identified.

## P16 Cutaneous T-cell lymphoma - What the radiologist needs to know and the pitfalls to avoid

### K Paramesh, D Dasgupta, C Goncalves

#### Guys and St Thomas' NHS Trust, UK, London

##### **Correspondence:** K Paramesh (akshatha_p@yahoo.com)

Learning objective:

To review imaging of extracutaneous manifestations of cutaneous lymphoma and identify potential pitfalls that create diagnostic uncertainty.

Content organisation:

Primary cutaneous lymphomas are a group of extranodal non-Hodgkin lymphomas confined to the skin with no extracutaneous disease at time of diagnosis. Mycosis fungoides (MF) and Sezary syndrome (SS) are the most common variants. MF is an indolent lymphoma and patients often have several years of skin lesions before the diagnosis is made. In later stages, lymphadenopathy and widespread visceral involvement occur, which significantly decrease 5-year survival, almost 0% with systemic spread.

However, radiological detection of extracutaneous disease is a diagnostic challenge. Imaging features of MF and SS can be non-specific and patients are susceptible to secondary malignancies, infections such as HIV and COVID-19 which create additional pitfalls.

We will review:

- Extracutaneous manifestations in lungs, central nervous system and abdominal viscera
- Nodal disease and dermopathic nodes
- Secondary malignancies
- Concurrent infection - HIV and COVID-19
- Differential diagnoses such as connective tissue disorders and cutaneous involvement of leukaemia
- Standardised assessment of extracutaneous disease burden

Conclusion:

Extracutaneous manifestations of cutaneous lymphoma significantly decrease patient survival but the imaging findings can be non-specific and confounding factors add to diagnostic uncertainty. Radiological demonstration of disease progression beyond the primary site is important as it has significant implications for management and patient survival.

## P17 Pre-operative diagnosis of extracapsular extension of prostate cancer using MRI derived semantic features

### Adalgisa Guerra, Liliana Parreira, Kris Maes, João Cassis, Filipe Caseiro-Alves, Rui Maio, Helena Mouriño

#### Radiology Department/Hospital da Luz Lisboa, Faculty of Sciences-University of Lisbon, Urology Department/Hospital da Luz Lisboa, Pathology Department/Hospital da Luz Lisboa, Faculty of Medicine-University of Coimbra, Nova Medical School-Nova University of Lisbon, Faculty of Sciences-University of Lisbon

##### **Correspondence:** Adalgisa Guerra (gisaguerra@gmail.com)

Aim: The purpose of this study was to assess the MRI morpho-structural (semantic) features and clinical findings for the diagnosis of extracapsular extension on pathology (pECE+) in patients with prostate cancer (PCa).

Methods: We performed a retrospective 3T MRI study comparing the clinical and MRI semantic features with pathology findings from 184 patients treated with Robotic Assisted Radical Prostatectomy (RARP). Rad-path correlation of the index lesion was performed. The study’s binary outcome variable was pECE status on prostate specimens. The covariates under consideration were clinical and MRI semantic features, according to PIRADS-v2, adding to these the capsular contact length (TCL) and the length of the index lesion. Simple and multiple logistic regression models were applied to explore the association between covariates and pECE.

Results: From the 184 patients, 25% were pECE+, and these had significantly (p-value<0,05) larger PSA density (mean 0.23 vs 0.19), higher index lesion size (mean 17.7 vs 12.9mm) and higher TCL (mean 20.3 vs 11.2) compared to patients without ECE on pathology (pECE-). Based on the multiple logistic regression model, TCL, capsular disruption, measurable ECE on MRI and GS ≥ 7(4+3) at biopsy were the impact risk factors for pECE+. The AUC was 0.906, providing high sensitivity (93%) but moderate specificity (70%).

Conclusion: Combination of high TCL, ECE on MRI and GS ≥7(4+3) on index lesion are sensitive discriminators for pre-operative diagnosis of pECE+.

## P18 Extranodal risk sites for Secondary CNS Lymphoma – Prognostication

### E.Musanhu; B.Sharma

#### The Royal Marsden Hospital, Sutton, UK

##### **Correspondence:** E.Musanhu (ed.musanhu@oxon.org)

Learning Objectives: To recognise the extranodal sites of lymphoma involvement which confer high risk for secondary CNS lymphoma.

Content organisation:

Lymphoma involving sites other than lymph nodes, spleen, thymus and Waldeyer’s ring is termed extranodal lymphoma. The higher the number of extranodal sites of involvement by diffuse large B-cell lymphoma, the higher the risk of secondary CNS lymphoma (SCNSL, a devastating complication of lymphoma, with poor outcomes), requiring prophylactic methotrexate. The testes, kidneys, adrenals, breasts are high risk extranodal sites for SCNSL. Paranasal, orbital, epidural involvement are also considered high risk by some series. Patterns of involvement are nuanced, a number of critical review sites apply on assessment of CT, PET/CT and MRI data in lymphoma patients. We present the imaging appearances of lymphoma on multimodal anato-functional imaging at the sites described above, and other extranodal sites, including the bone marrow and the myriad of lung lymphoma appearances. Prognostic scores for extranodal involvement and SCNSL are described. The nuanced appearances of lymphoma response at extranodal sites, including the newly described concepts of Indeterminate Response (IR1,2,3; the LyRIC modification), and the Deauville Score of ‘X’, significantly under recognised in clinical and research trial practice are described.

Conclusions:

Radiologists should explicitly state (yes/no) whether the kidneys, adrenals and liver are involved in all diffuse large B-cell lymphoma CT and PET reports. The number of extranodal sites involved should be stated in all imaging report conclusions at baseline for patients with diffuse large B-cell lymphoma.

## P20 Breast Cancer Metastasis to the Sacral Nerve

### Ahmad NA, Ibrahim N, Zulkilpully FN, Azmi ND, Hamdan MF, Awang MA

#### Institut Kanser Negara Malaysia, Hospital UITM Malaysia, Hospital Pengajar Universiti Putra Malaysia

##### **Correspondence:** Ahmad NA (azlinahmad@gmail.com)

1. To describe an unusual site of breast cancer metastasis in the spine, namely to the sacral nerve.
2. To discuss CT and MR features of benign versus malignant (primary or secondaries) sacral spinal lesions.
3. To highlight the challenges faced by clinicians in diagnosing and managing metastatic disease where tissue sample of the metastatic lesion could not be obtained.

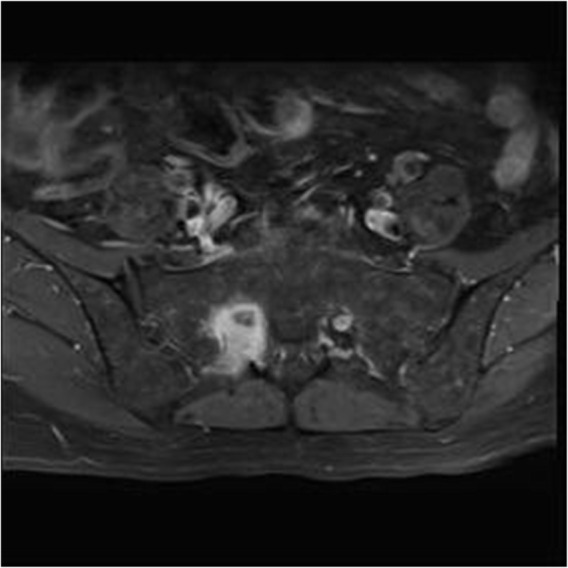

## P21 Adrenal masses - diagnostic challenges in an oncology multi-disciplinary meeting

### K Paramesh, H Verma, A Jacques

#### Guys and St Thomas' NHS Trust

##### **Correspondence:** K Paramesh (akshatha_p@yahoo.com)

**Aim:** Adrenal masses - diagnostic challenges in an oncology multi-disciplinary meeting

K Paramesh*, H Verma, A Jacques

Guys and St Thomas’ NHS Trust, London, UK

Learning objectives

To build a toolkit for how to approach an indeterminate adrenal lesion in a patient with known malignancy

To review the spectrum of adrenal lesions found in common clinical practice

Content organisation

Widespread use of imaging has significantly increased the detection of incidental adrenal lesions, which are largely benign, non-functioning adenomas. Even in patients with a known cancer, >90% lesions are benign. Nonetheless, accurately differentiating a benign adrenal lesion from a malignant one is crucial in oncology patients as it has significant implications for management and prognosis.

We will review the tools a radiologist can use in the setting of an oncology MDM for characterisation and risk-stratification of indeterminate adrenal masses. In addition, primary adrenal lesions such as phaeochromocytomas and functioning adenomas have significant implications for peri-operative management and patient outcome. Therefore, correlation of imaging with biochemical assessment of functionality and with histology prior to resection is important.

We will review:

Adenomas and washout characteristics

Haemangiomas

Myelolipomas

Phaeochromocytomas including in genetic conditions and the role of Ki-67

Metastases

Adrenocortical carcinomas

Conclusion

Adrenal lesions are common and can present diagnostic challenges due to overlap of imaging features between benign and malignant lesions. Detection, characterisation (if possible) and risk stratification based on radiological appearances is important, especially in oncology patients where it has significant implications on patient management.

## P22 Role of MRI in detecting Cervical Cancer Recurrence after Concurrent Chemoradiotherapy (CCRT)

### Ibrahim N, Ahmad NA, Zulkilpully FN, Yahya H, Zulkifli NS

#### Institut Kanser Negara Malaysia

##### **Correspondence:** Ahmad NA (azlinahmad@gmail.com)

Learning objective

To describe MRI characteristics of cervical cancer recurrence, and identify key MRI sequences used in follow up of cervical cancer post treatment

Content organisation

Cervical cancer is the second most common cancer in females, and ranked ninth overall highest cancer in Malaysia. The majority of cervical cancer patients in Malaysia are diagnosed at Stage II and Stage III. Post primary treatment, the majority of recurrences are detected within two years of treatment completion, with slightly more than half recurring in the pelvis. Most of the patients (65%) are symptomatic at recurrence. We will review the incidence of cervical cancer recurrence in Malaysia, including:

• The common timeline of cervical cancer recurrence after treatment completion

• Factors that may affect cervical cancer recurrence such as histopathology of primary lesion and stage at diagnosis We will discuss MRI features of cervical cancer recurrence, including: • Imaging timeline for reassessment of cervical cancer post CCRT

• Correlation of MRI features with clinical findings and histopathology

• Differentiating between post treatment changes and local recurrence

• Key MRI sequences used to detect recurrence at early stage

• Use of dynamic contrast enhancement sequence in detecting small recurrences

Conclusion

MRI is helpful in identifying cervical tumour recurrence after CCRT. Routine follow up with MRI pelvis is recommended post treatment in order to detect early recurrence especially among asymptomatic patients.

